# Overall survival after treatment for metastatic uveal melanoma: a systematic review and meta-analysis

**DOI:** 10.1097/CMR.0000000000000575

**Published:** 2019-01-16

**Authors:** Elina S. Rantala, Micaela Hernberg, Tero T. Kivelä

**Affiliations:** Departments of a Ophthalmology, Ocular Oncology Service; b Oncology, Comprehensive Cancer Centre, Helsinki University Hospital and University of Helsinki, Helsinki; c Department of Ophthalmology, Etelä-Pohjanmaa Central Hospital, Seinäjoki, Finland

**Keywords:** melanoma, meta-analysis, metastasis, survival analysis, survival, systematic review, treatment, uveal melanoma, uveal neoplasms

## Abstract

The overall survival (OS) of patients with metastatic uveal melanoma is short, the evidence for effectiveness of treatments is limited, and no consensus on the choice of treatment exists. We aimed to advance interpretation of OS as an outcome by pooling peer-reviewed data. The design is a systematic review and meta-analysis. We searched PubMed from 1 January 1980, to 29 March 2017, for articles reporting patient-level survival in Kaplan–Meier or numerical form. We digitized survival graphs, pooled individual survival times, calculated median OS by treatment modality, and compared each modality by the log-rank test and Cox regression using conventional chemotherapy (CHT) as a reference. Individual-level data were obtained from 78 articles with 2494 patients. The median OS across all treatment modalities was 1.07 years (range: 0.59–2.50 years). Pooled OS reported after isolated hepatic perfusion [median OS: 1.34 years; hazard ratio (HR): 0.92, 95% confidence interval (CI): 0.87–0.97, *P* = 0.0040], immunoembolization (median OS: 1.63; HR: 0.97, 95% CI: 0.95–1.00, *P* = 0.0080), and surgery (median OS: 1.43; HR: 0.94, 95% CI: 0.92–0.96, *P* < 0.0001) was longer, and after checkpoint inhibitor shorter (median OS: 0.59; HR: 1.13, 95% CI: 1.06–1.20, *P* < 0.0001) than after CHT (median OS: 0.91 years), but subject to identifiable confounding factors. OS following other modalities did not differ from CHT. Reported OS was unassociated with the decade of publication, but depended on the percentage of first-line treated patients. Our results suggest no clinically significant difference in OS by treatment modality or decade. Most of the difference in reported OS likely is attributable to surveillance, selection, and publication bias rather than treatment-related prolongation. Our pooled data provide benchmarks for future trials.

## Abstract 2

Supplemental Digital Content is available in the text.

## Introduction

Uveal melanoma is the most common primary malignant intraocular tumour in adults [1]. The incidence varies by age, ethnicity and latitude from 0.1 to 8.6 per million [2,3]. More than half result in metastases [4] and thereafter the median overall survival (OS) historically is less than 6 months [5]. The liver remains the only site of metastasis in half of the patients [5,6]. Improvements in managing the primary tumour have not translated into decreased metastasis rate or longer survival [1,7].

Over the last decade, considerable development has occurred in targeted therapy for metastatic cutaneous melanoma [8], but new treatments have not shown equal survival benefits in metastatic uveal melanoma [9,10] and no standard first-line treatment exists. Because of the small number of patients, few randomized trials have been conducted [11–14]. We aimed to advance the interpretation of survival outcomes by extracting individual-level OS data from peer-reviewed articles by digitizing Kaplan–Meier curves and by pooling data for comparison.

## Patients and methods

### Search strategy and selection criteria

We specified the methods and inclusion criteria of our systematic review and meta-analysis according to the PRISMA 2009 guidelines [15] and performed a literature search without language restrictions to identify all articles on metastatic uveal melanoma in peer-reviewed journals from 1 January 1980, through 29 March 2017.

We searched PubMed with the terms uveal melanoma, choroidal melanoma, ciliary body melanoma, ciliochoroidal melanoma, iridociliary melanoma, iris melanoma, intraocular melanoma and ocular melanoma, combined with metast* or stage IV and treatment (PubMed search strategy, Supplemental digital content 1, *http://links.lww.com/MR/A107*). One author (E.S.R.) reviewed the titles and abstracts to confirm the subject was treatment of metastatic uveal melanoma. Articles on animal models, laboratory investigations, imaging, primary or locally recurrent tumour, prognosis, staging, quality-of-life, reviews and case reports were excluded. Studies with less than three patients were excluded to limit most obvious publication bias.

Two authors (E.S.R. and T.T.K.) reviewed the full text of relevant articles. Studies that included primary cutaneous or mucosal melanoma were excluded unless patients with uveal melanoma were reported separately. If the primary was designated ocular, we e-mailed the first author to exclude conjunctival melanomas. Of 17 authors, 14 responded and we categorized the publications accordingly; the remaining articles were excluded. If it was uncertain whether patients in any two articles overlapped, we excluded the article with fewer patients. Finally, we checked the reference lists against our search and archives to identify additional ones.

### Data analysis

Our endpoint was OS. If the article referred to RECIST criteria [16], we assumed OS to begin from initiating study treatment until censoring or death. If multiple definitions were provided, we chose the one corresponding to the Kaplan–Meier graph. We tabulated the author, publication year, treatment, study design, number of patients, first-line treatments and previous surgeries, OS with definition, and geographical origin of each publication (see Supplementary Table, Supplemental digital content 2, *http://links.lww.com/MR/A108*, which summarizes the characteristics of included studies on treatment for metastatic uveal melanoma). If the median OS was unreported, we tabulated it from our digitized Kaplan–Meier curve; in case the reported OS differed from our digitized one, we suggest an explanation.

We extracted the individual-level survival times directly when reported in the text or a table. In general, patient-level data were not tabulated and we scanned the Kaplan–Meier curve(s) to TechDig (version 2.0.0.1; shareware by Ronald B. Jones, Mundelein, Illinois, USA) to manually obtain the survival time corresponding to each step for deaths and tick for censored events. If the latter were not displayed, but the at-risk table was provided, the at-risk reduction minus deaths was taken to be the number of censored events during each interval, which we assigned to its midpoint [17]. If the at-risk table was absent, we calculated the total number of censored events from the total number of patients and deaths. We then transferred the digitized survival times to Stata (version 13; Stata Corp., College Station, Texas, USA), plotted the survival curve and overlaid it as a transparent layer over the appropriately resized published graph in Paint Shop Pro X6 (Corel Corp., Ottawa, Canada). We visually identified intervals with censored event(s), assigned these events by trial-and-error and repeated this process until the survival graphs matched. We verified other digitized Kaplan–Meier graphs similarly. The data are available from the Zenodo Digital Repository (*https://doi.org/10.5281/zenodo.1490563*).

We prospectively identified the main treatment modalities: conventional chemotherapy (CHT), chemoimmunotherapy (CIT), hepatic intra-arterial chemotherapy (HIA), transarterial chemoembolization (TACE), isolated hepatic perfusion (IHP), checkpoint inhibitor (CPI), protein kinase inhibitor, selective internal radiation therapy, immunoembolization (IE) and immunosuppressant. Moreover, we found data on liver-directed thermotherapy (laser-induced thermotherapy and stereotactic radiofrequency ablation), vaccine and surgery. Best supportive care was excluded [18–21].

We compared individual studies on each treatment modality to assess heterogeneity, and then compared studies within each modality according to the agents used, if applicable. We next compared each treatment modality, in aggregate, against CHT and, finally, limited our analysis to first-line treatments whenever possible. We tabulated the median OS with the 95% confidence interval (CI), plotted comparative Kaplan–Meier graphs and used the log-rank test and univariable Cox proportional hazards regression analysis to compare modalities, setting significance at 0.05.

## Results

**Fig. 1:**
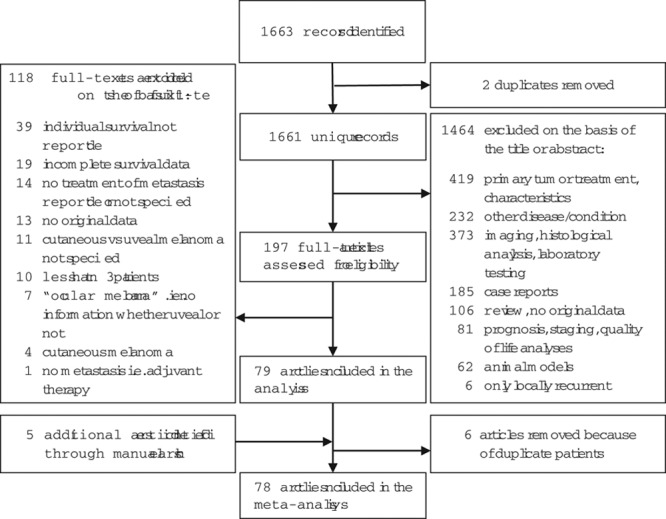
Study flow chart.

Our search identified 1663 records on metastatic uveal melanoma. Review of abstracts found 197 articles on the treatment of at least three patients (Fig. 1; see Supplementary Table for exact patient number for each article, Supplemental digital content 2, *http://links.lww.com/MR/A108*, which summarizes the characteristics of included studies on treatment for metastatic uveal melanoma). From the reference lists and our archives, we found five additional articles. We excluded six articles because of uncertainty in terms of whether they shared patients with other publications (Supplementary Text, Supplemental digital content 3, *http://links.lww.com/MR/A109*, which summarizes the excluded studies). Seventy-eight of the 196 articles contained data digitizable for a pooled Kaplan–Meier graph.

Of the 78 articles, 37 (47%) were prospective and 41 (53%) were retrospective (see Supplementary Table, Supplemental digital content 2, *http://links.lww.com/MR/A108*, which summarizes the characteristics of included studies on treatment for metastatic uveal melanoma). OS was reported from the diagnosis of metastases in eight (10%), enrolment in six (8%), initiation of study treatment in 59 (76%) and was undefined in five (6%) publications. Multiple definitions were adopted in 11 articles. Nineteen (24%) studies were funded totally or partially by industry, the authors reported a conflict of interest with the industry, or both. In 36 (46%) studies, the industry was not involved, and in 23 (29%), this information was unavailable.

Altogether, we included 2494 patients, ranging from three to 171 by study (Supplementary Table, Supplemental digital content 2, *http://links.lww.com/MR/A108*); 478 were censored observations. Of the latter, 182 (38%), 305 (64%) and 379 (79%) had occurred by 1, 2 and 3 years, respectively. The median OS was 1.07 years (95% CI: 1.00–1.13), but the survival rate decreased rapidly from 52% (95% CI: 50–54) at 1 year to 25% (95% CI: 23–26) at 2 years, and 13% (95% CI: 12–15) at 3 years (Table 1). The median OS on the basis of publications that reported first-line treatments was 1.03 years (95% CI: 0.95–1.14) for 510 patients, and the survival rate was 51% (95% CI: 47–55), 21% (95% CI: 18–25) and 10% (95% CI: 7–13) at 1, 2 and 3 years, respectively (see Table 1 for data by therapeutic modality).

**Table 1:**
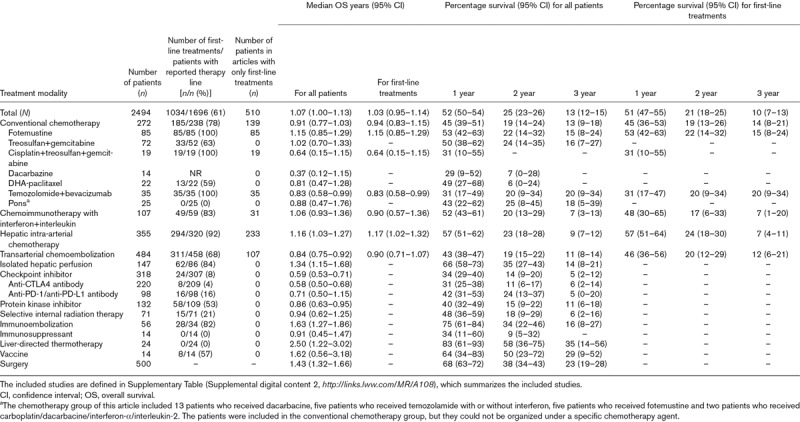
Treatment modalities with number of patients, number of first-line treatments, median overall survival and 1-, 2- and 3-year survival

**Fig. 2:**
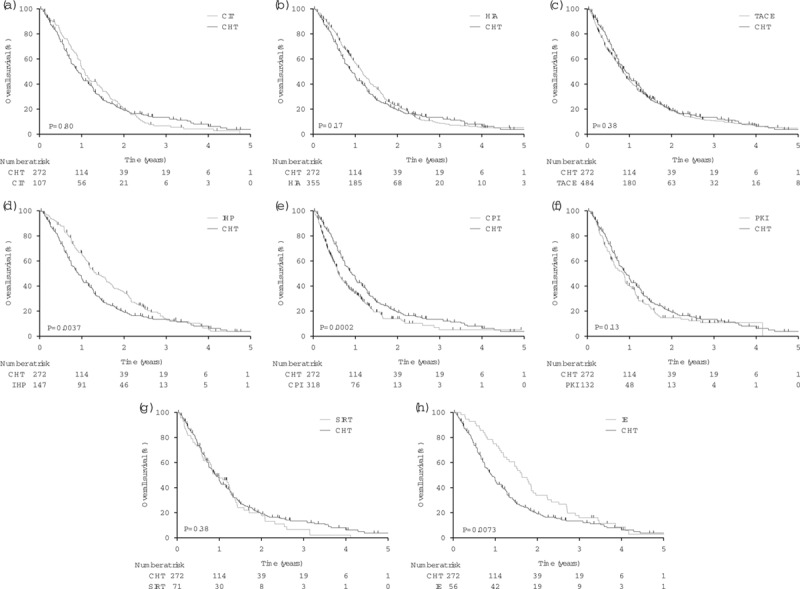
Kaplan–Meier plots (a–h) of overall survival after metastatic uveal melanoma, pooled data for each treatment modality administered to more than 50 patients, compared against conventional chemotherapy. *P* values were calculated using the log-rank test. CHT, conventional chemotherapy; CIT, chemoimmunotherapy; CPI, checkpoint inhibitor; HIA, hepatic intra-arterial chemotherapy; IE, immunoembolization; IHP, isolated hepatic perfusion; IS, immunosuppressant; LDT, liver-directed thermotherapy; PKI, protein kinase inhibitor; SIRT, selective internal radiation therapy; TACE, transarterial chemoembolization.

**Fig. 3:**
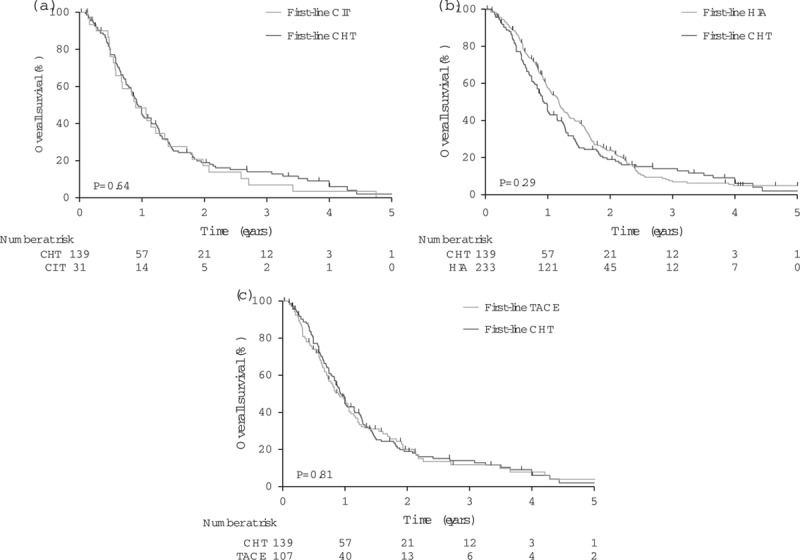
Kaplan–Meier plots (a–c) of overall survival after metastatic uveal melanoma, pooled data for each treatment modality, restricted to first-line treatments, compared against conventional chemotherapy. *P* values were calculated using the log-rank test. CHT, conventional chemotherapy; CIT, chemoimmunotherapy; HIA, hepatic intra-arterial chemotherapy; TACE, transarterial chemoembolization.

We provide Kaplan–Meier graphs by article for each treatment modality (Supplementary Fig. S1, Supplemental digital content 4, *http://links.lww.com/MR/A110*) and CHT agent (Supplementary Fig. S2, Supplemental digital content 4, *http://links.lww.com/MR/A110*) to show heterogeneity between articles, and graphs for each modality against CHT for all patients (50 patients or more, Fig. 2; fewer than 50 patients; Supplementary Fig. S3, Supplemental digital content 4, *http://links.lww.com/MR/A110*) and for first-line treatments, as available (Fig. 3). OS was comparable to that with CHT for CIT, HIA, TACE, protein kinase inhibitor and selective internal radiation therapy (*P* = 0.13–0.80). We also show components of survival for studies that report interval from diagnosis of metastases to the initiation of study treatment; this interval varied widely and adds to heterogeneity (Supplementary Fig. S4, Supplemental digital content 4, *http://links.lww.com/MR/A110*). IHP [hazard ratio (HR): 0.92, *P* = 0.0040] and IE (HR: 0.97, *P* = 0.0080) were associated with longer OS, and CPI (HR: 1.13, *P* < 0.0001) with shorter OS than CHT (Table 2 and Fig. 2). Patient-level data on first-line treatments were unavailable for these treatment modalities. However, sensitivity analysis showed that the difference in OS after IHP depends solely on the largest of the six studies in which OS was exceptionally long (Supplementary Fig. S5, Supplemental digital content 4, *http://links.lww.com/MR/A110*) [22], whereas only about 8% of CPI treatments in the nine studies aggregated were first-line (Supplementary Table, Supplemental digital content 2, *http://links.lww.com/MR/A108*). The IE data derived from a single-centre phase I and a subsequent phase II trial [12,23].

**Table 2:**
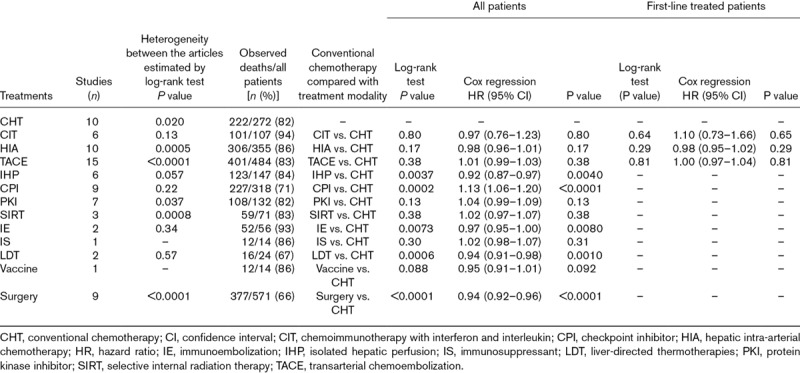
Heterogeneity between publications reporting treatment for metastatic uveal melanoma and comparison of pooled overall survival data by treatment modalities against conventional chemotherapy

To determine whether the treatment outcome has improved over time, we compared the median OS according to publishing decade. The number of patients was 1994 after exclusion of surgery. From the 1980s to the 2010s, OS was 0.92 (95% CI: 0.66–1.50; 2% of patients), 1.16 (0.93–1.51; 2%), 1.03 (0.96–1.12; 26%) and 0.91 (0.85–0.99; 70%) years, showing no improvement (log-rank test for trend *P* = 0.66; Supplementary Fig. S6, Supplemental digital content 4, *http://links.lww.com/MR/A110*).

To estimate bias from treatment line, we categorized articles according to the percentage of patients who received first-line treatment. Studies with exclusively first-line treatments had the longest and those with no first-line treatments had the shortest pooled OS; survival times associated with increasing percentages of first-line treated patients were intermediate, but not ordered logically (log-rank test for trend *P* < 0.0001; Supplementary Fig. S7, Supplemental digital content 4, *http://links.lww.com/MR/A110*; Table 2). When the number of first-line treatments was unreported, OS was longest, a finding not explained either by more frequent reporting of OS from diagnosis versus enrolment or study treatment (6 vs. 26% for all first-line treatments group) or by more frequent liver-targeted therapies (67 vs. 67%, respectively).

## Discussion

In our meta-analysis, the median OS after metastatic uveal melanoma was 1.07 years for 2494 patients, ranging from 0.84 to 1.34 years for main treatment modalities other than CPI (0.59) and IE (1.63). These differences likely would diminish if the analysis could be limited to first-line treatments [24], but such patient-level data were available only for CHT, CIT, HIA and TACE. The median OS was longer (1.43) than average for surgically treated patients, but only selected ones are eligible because of frequent widespread hepatic metastases, even when surveilled intensively [19].

Traditionally, phase II trials have reported the objective response rate as the endpoint. A review of 841 patients from 40 articles of metastatic uveal melanoma analysed the objective response rate, but not OS [9]; of the 78 articles in our meta-analysis, 70% were not available for that study. OS arguably is a more meaningful outcome because, for the patient, disease stabilization or even slower progression matters [25]. We chose OS as the endpoint also because it was most frequently available at the patient level and least subject to interpretation. The past 25 years in Uveal Melanoma: a Meta-Analysis Study, a so far unpublished meta-analysis of the International Rare Cancers Initiative, aims to pool original data of 970 patients from 29 phase II trials to establish benchmarks of 6-month progression-free survival and 1-year OS [26,27]. Interim subgroup analyses based on 677 and 463 patients are available as abstracts without pooled OS [26,27]. Another review tabulated the median OS from 36 articles without pooling data [10].

Most studies that we included were retrospective and had small sample sizes. A consequent limitation is the high level of heterogeneity from publication and selection bias and variable case mix. Some metastases were detected by surveillance and others after symptoms developed; some patients were treatment-naive and others had been pretreated heavily. We made the latter bias apparent by tabulating the percentage of first-line treatments. Resection of metastases, even when partial, may prolong survival [19,28]. We highlighted this bias by reporting the number of patients who underwent a previous resection. Other sources of bias including performance status, metastasis sites and burden, and liver function were reported variably and not at the patient level, and we could not adjust for them. However, bias should become averaged when data from an increasing number of studies are pooled.

Heterogeneity was evident even in the CHT group used as a comparison base. All patients treated with fotemustine derived from the largest prospective randomized study, and all treatments were first-line [11], whereas patients treated with treosulfan plus gemcitabine came from four publications and 43% of treatments were higher than first line [29–32]. Nevertheless, we detected no OS difference between these regimens (*P* = 0.94).

With respect to treatments other than CHT, a prospective multicentre study [33] analysed the efficacy of BOLD chemotherapy plus recombinant interferon α-2b, a form of CIT, because of encouraging pilot reports [34,35], but did not confirm its efficacy. Our pooled data from five CIT studies confirmed no difference compared with CHT.

Administration of chemotherapeutic agents to the hepatic artery with or without blocking the blood supply of the metastases is logical considering that the liver typically is the first and the only site of metastasis [5,6]. IHP with melphalan, sometimes with an additional agent, was associated with a longer OS than CHT. It is offered in dedicated centres, one of which [22] was responsible for the difference, sometimes with considerable complications, weighted mean mortality 6% [22].

Studies with no first-line treatments expectedly reported the shortest pooled median OS, reflecting the salvage nature of the treatment. We addressed bias from varying percentages of first-line treatments by tabulating the percentages and analysing articles accordingly. Although survival was intermediate for all combinations of first-line and higher-line treatments, it did not logically decrease with decreasing percentage of first-line treatments, indicating a complicated source of bias that included variable time from diagnosis of metastases. Nevertheless, the rarity of first-line treatments explained worse survival after CPI. Analysis of the OS according to publishing decade found no improvement over time. No valid comparison of immunotherapy combined with other treatments was possible and more trials using immunotherapy backbone are needed.

Different methodologies in original publications posed a challenge. The method to calculate the median OS was unreported occasionally. Kaplan–Meier graphs often lacked at-risk tables and censored events. Assigning censored events to the midpoint of each interval, whilst recreating each curve, potentially introduced bias when we pooled data from several studies, and their censored events and deaths became intermixed. This bias should become less influential with increasing numbers of pooled studies. On the basis of the difficulties in extracting data, we propose guidelines for reporting on treatment for metastatic uveal melanoma (see Supplementary Text, Supplemental digital content 5, *http://links.lww.com/MR/A111*, which proposes guidelines for future reporting of studies on treatment for metastatic uveal melanoma). As previously done for cutaneous melanoma [36], we provide a benchmark dataset to facilitate future trial design and analysis (see Supplementary Text, Supplemental digital content 6, *http://links.lww.com/MR/A112*, which describes overall survival benchmark; and the Supplementary Excel file, Supplemental digital content 7, *http://links.lww.com/MR/A113*, which includes the numerical data to calculate the historical survival curve). We acknowledge that our meta-analysis cannot replace prospective randomized studies.

## Conclusion

Despite suggestions in uncontrolled studies [37–39], patient-level data aggregated from peer-reviewed articles provide no compelling evidence for a longer median OS for patients with metastatic uveal melanoma by any treatment modality, confirming traditional reviews [9,10] and rare previous randomized trials [11–14]. The difference in OS between nonrandomized studies likely is attributable to surveillance, selection and publication bias rather than treatment-related prolongation.

## Acknowledgements

The authors thank all previous authors who provided additional data to us.

Elina S. Rantala has received a grant from the Evald and Hilda Nissi Foundation, Finland, and a grant from Etelä-Pohjanmaa Central Hospital, Finland. Tero T. Kivelä has received a grant from Cancer Foundation Finland, a grant from Sigrid Juselius Foundation, Finland, and a grant (grant no.: TYH2017218) from the Helsinki University Hospital Research Fund.

## Conflicts of interest

Micaela Hernberg reports personal fees from Amgen, BMS, Incyte, MSD, Novartis, and Roche outside the submitted work. For the remaining authors, there are no conflicts of interest.

## Supplementary Material
