# Supplementary material for: Overall survival after treatment for metastatic uveal melanoma: a systematic review and meta-analysis
Source: Melanoma Res. 2019 Jan 16;29(6):561–8. doi: 10.1097/CMR.0000000000000575 (PMC6887637; doi:10.1097/CMR.0000000000000575)
Supplement: Supplementary file 1 [file mr-29-561-s001.pdf]

Supplemental digital content 1.pdf - PubMed search strategy

PubMed search:

(uveal melanoma OR choroidal melanoma OR ciliary body melanoma OR ciliochoroidal melanoma OR iridociliary melanoma OR iris melanoma OR intraocular melanoma OR ocular melanoma) AND (metast* OR stage IV) AND (treatment) AND (“1980/01/01” [PDAT] : “2017/03/29” [PDAT]).

The search was performed on March the 29th 2017.
