# Supplementary material for: Overall survival after treatment for metastatic uveal melanoma: a systematic review and meta-analysis
Source: Melanoma Res. 2019 Jan 16;29(6):561–8. doi: 10.1097/CMR.0000000000000575 (PMC6887637; doi:10.1097/CMR.0000000000000575)
Supplement: Supplementary file 2 [file mr-29-561-s002.pdf]

Supplemental digital content 2.pdf - Summary of the characteristics of all included studies on treatment for metastatic uveal melanoma

Intervention	Study	Publication year	Study design	Number of uveal melanoma patients	Number of first-line treatments (%)	Number of surgically treated patients (%)	Median time from metastasis to treatment months, (range)	OS definition	Median OS, published Kaplan-Meier estimate months, (95% CI)	Median OS, digitised Kaplan-Meier estimate months, (95% CI)	Region(s)
**Conventional chemotherapy (CHT)**											
Dacarbazine	Carling [1]	2015	Retrospective CCS	14	NR	NR	2.5 (0.6-4.7)	Treatment	4.6	4.5 (1.4-13.7)	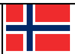
Gemcitabine + treosulfan	Corrie [2]	2005	Prospective NRCS phase I	5	4 (80%)	0 (0%)	NR	NR	12.2	12.2 (4.6-ND)	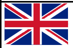
Docosahexaenoic acid-paclitaxel	Homsi [3]	2010	Prospective NRCS phase II, open label	22	13 (59%) NC	NR	NR	Enrollment	9.8	9.8 (5.6-15.4)	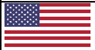
Fotemustine	Leyvraz [4]	2014	Prospective randomised trial EORTC multicentre	85	85 (100%)	0 (0%)	NR	Enrollment	13.8 (10.2-17.2)	13.8 (10.3-15.5)	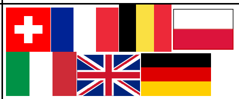
Gemcitabine + treosulfan	Pföhler [5]	2003	Retrospective case series	14	1 (7%)	0 (0%)	NR	Enrollment	14 (12-31)	13.8 (4.1-ND)	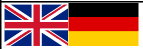
Temozolomide + bevacizumab	Piperno-Neumann [6]	2016	Prospective trial phase II	35	35 (100%)	0 (0%)	NR	Treatment	10 (8-15)	10.0 (6.9-11.9)	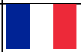
Multiple*	Pons [7]	2011	Retrospective CCS	25	0 (0%)	0 (0%)	NR	NR	10.83 (5.35-16.30)	10.5 (5.6-21.1)	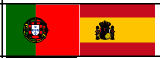
Gemcitabine + treosulfan + cisplatin	Schmittel [8]	2005	Prospective NRCS phase II	19	19 (100%) NC	NR	NR	Treatment	7.7 (1.9-13.8)	7.7 (1.8-13.9)	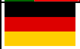
Gemcitabine + treosulfan 2500 or 3000 mg/m^2	Schmittel [9]	2005	Prospective NRCS phase II	14	12 (86%) NC	NR	NR	Treatment	6.0 (4-8)	5.9 (2.4-8.0)	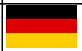
Gemcitabine + treosulfan 3500 or 4000 mg/m^2				19	16 (84%) NC	NR	NR	Treatment	9.0 (0-18)	8.9 (4.4-24.9)	
Gemcitabine + treosulfan	Terheyden [10]	2004	Prospective NRCS phase II	20	8 (40%)	0 (0%)	NR	Treatment	NR	17.0 (9.0-31.0)	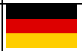

*Thirteen patients received dacarbacine, five received temozolomide with or without interferon, five received fotemustine, two received carboplatin/dacarbacine/interferon-alpha/interleukin-2. Because of multiple different therapies the patients were included in the conventional chemotherapy group but could they could not be organised under a specific chemotherapy agent.

**Chemoimmunotherapy (CIT)**											
Fotemustine IA/IV + IL2+ IFN-alpha2	Becker [11]	2002	Prospective NRCS	48	NR	NR	NR	Treatment	12 (11.4-12.8)[*]	14.1 (11.7-19.6)	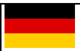
BOLD + IFN-alpha2b	Kivelä [12]	2003	Prospective nonrandomised trial phase II EORTC multicentre	24	24 (100%)	0 (0%)	NR	Treatment	10.6 (6.9-16.4)	10.4 (5.9-14.6)	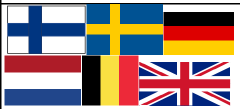
BOLD + leucocyte IFN	Pyrhönen [13]	2002	Prospective nonrandomised trial phase II	22	18 (82%)	0 (0%)	NR	Treatment	12 (8-22)	12.4 (8.2-22.2)	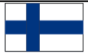
Thalidomide + IFN-alpha2b	Solti [14]	2007	Prospective NRCS pilot study	6	0 (0%)	NR	NR	Treatment	NR	3.7 (0.9-ND)	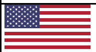
Fotemustine IV + IL2 + IFN-alpha2b	Terheyden [15]	1998	Retrospective case series	3	3 (100%)	0 (0%)	2 (2-4)	Treatment	NR	41.0 (10.0-ND)	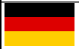
Dacarbazine + IFN-alpha2a + bevacizumab	Vihinen [16]	2010	Prospective trial phase II	4	4 (100%)	0 (0%)	NR	Treatment	NR	10.8 (6.5-ND)	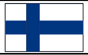
**Hepatic intra-arterial chemotherapy (HIA)**											
Carboplatin	Cantore [17]	1994	Prospective NRCS phase II	8	5 (63%)	NR	NR	Treatment	15[†]	12.0 (7.0-20.0)	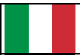
Fotemustine	Egerer [18]	2001	Prospective NRCS pilot study	7	5 (71%)	0 (0%)	2 (1-10)	Treatment	18[‡]	20.0 (3.0-ND)	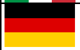
Fotemustine or carboplatin	Farolfi [19]	2011	Retrospective case series	18	NR[§]	NR	NR	Treatment	21 (8-39)	21.2 (7.4-ND)	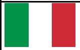
Melphalan<br><br>Melphalan and additional agents[**]	Heusner [20]	2011	Retrospective CCS	38<br><br>23	43 (70%) NC	NR<br><br>NR	3.3 (0.5-2), mean	Treatment	10.3 (5.01-15.59)<br><br>8.7 (8.07-9.32)	10.2 (4.6-16.4)<br><br>8.5 (5.5-13.3)	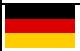
Fotemustine	Leyvraz [4]	2014	Prospective randomised trial EORTC multicentre	86	86 (100%)	0 (0%)	NR	Enrollment	14.6 (10.2-15.4)	14.6 (10.2-17.2)	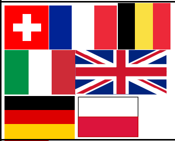
Fotemustine	Leyvraz [21]	1997	Prospective NRCS	31	31 (100%) NC	NR	NR	Diagnosis	14	11.8 (6.7-18.6)	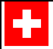

[*] The Kaplan-Meier graph did not have steps and possibly because of resultant difficulty in digitizing the median survivals differ.

[†] Median survival is 12 months when calculated from Table 1; the reported median survival of 15 months appears to correspond to mean survival [(7+8+8+12+16+18+20+29)/8=15 months].

[‡] The reported median survival of 18 months appears to be arithmetically calculated and not a Kaplan-Meier estimate.

[§] Reported for all patients, not specifically for uveal melanoma patients.

[**] Dacarbazine, doxorubicin, fotemustine, gemcitabine, MMC.

Cisplatin + vinblastine + dacarbazine	Melichar [22]	2009	Retrospective case series	10	7 (70%)	0 (0%)	1.4 (0.5-9.5)	Treatment	16	16.0 (5.0-19.0)	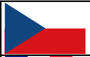
Fotemustine or dacarbazine + cisplatin, after biopsy[*]	Salmon [23]	1998	Prospective nonrandomised comparative case series	16	16 (100%)	0 (0%)	NR	NR	NR	6.2 (1.0-10.1)	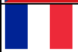
Fotemustine	Siegel [24]	2007	Retrospective case series	18	NR	0 (0%)	2 (6-63)	Treatment	22	22.0 (6.1-31.8)	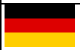
Fotemustine[†]	Peters [25]	2006	Retrospective case series	101	101 (100%) NC	39 (39%)[‡]	1.9 (0.1-45)	Diagnosis	15 (12.1-17.6)	14.9 (13.3-19.1)	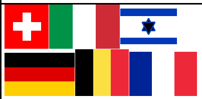
**Transarterial chemoembolisation (TACE)**											
Cisplatin w/wo polyvinyl sponge[§]	Agarwala [26]	2004	Prospective randomised trial phase I/II	19	13 (68%)	0 (0%)	NR	NR	8.5	8.4 (2.9-16.0)	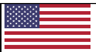
Irinotecan-eluting beads	Carling [1]	2015	Retrospective CCS	14	11 (79%)	1 (7%)	4.1 (1.2-12.7)	Treatment	9.4	9.4 (2.7-14.2)	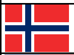
Cisplatin + doxorubicin + MMC + gelatin sponge	Dayani [27]	2009	Retrospective case series	21	17 (81%)	1 (5%)	NR	Treatment	NR	5.1 (3.3-8.2)	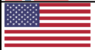
Fotemustine + polyvinyl alcohol particles	Edelhauser [28]	2012	Retrospective case series	21	2 (10%)	0 (0%)	NR	Diagnosis	NR	28.8 (9.9-ND)	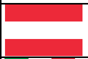
Irinotecan-eluting beads	Fiorentini [29]	2009	Prospective trial phase II	10	0 (0%)	0 (0%)	44 (24-84)	Treatment	NR	ND[**]	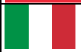
BCNU + gelatin sponge	Gonsalves [30]	2015	Retrospective case series	50[††]	50 (100%)	0 (0%)	NR	Treatment	7.1	7.1 (4.3-8.0)	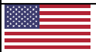
Cisplatin[‡‡] + gelatin sponge or polyvinyl alcohol particles	Gupta [31]	2010	Retrospective case series	125	82 (66%)	NR	7 (1-122), mean	Treatment	6.7 (4.9-8.5)	6.8 (4.8-8.9)	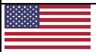
Cisplatin or carboplatin + polyvinyl alcohol particles	Huppert [32]	2010	Prospective NRCS pilot study	14	8 (57%)	1 (7%)	4.5 (1-38)	Treatment	11.5	12.0 (9.0-22.0)	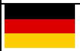
Cisplatin + polyvinyl sponge	Mavligit [33]	1988	Retrospective case series	30	19 (63%)	0 (0%)	NR	Diagnosis	11 (9-18)	11.1 (8.0-18.0)	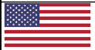
BCNU + gelatin sponge	Patel [34]	2005	Prospective trial phase II	30	24 (80%)	0 (0%)	NR	Treatment	5.2	5.0 (2.9-9.8)	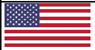

[*] Patients received at least two months fotemustine or dacarbazine and cisplatin after biopsy.

[†] In addition, 38 patients underwent debulking surgery at the time of catheter placement. In the Kaplan-Meier plot 100 patients were depicted.

[‡] As prior treatment, 39 patients had undergone surgery; the prior treatment of 11 patients was unknown.

[§] Of 19 patients, ten were treated with TACE.

[**] Median overall survival was not reached, i.e. cumulative survival plot did not fall below 50%.

[††] Fifty patients met the inclusion criteria but 49 patients are included in the Kaplan-Meier analysis.

[‡‡] Two patients additionally received paclitaxel and one patient doxorubicin + MMC.

Fotemustine or cisplatin + starch microspheres	Schuster [35]	2010	Retrospective case series	25	0 (0%)	NR	9 (2-24)	Treatment	6 (5-7)	5.8 (4.9-10.8)	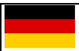
Cisplatin + gelatin sponge	Shibayama [36]	2017	Retrospective case series	29	27 (93%)	1 (3%)	NR	Treatment	23	23.0 (13.0-27.0)	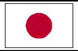
CPT-11 charged microbeads	Valpione [37]	2015	Retrospective CCS	58*	58 (100%)	0 (0%)	NR	Treatment	15.5†	16.5 (12.8-23.2)	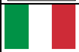
Bland embolisation + gelatin sponge	Valsecchi [38]	2015	Prospective randomised trial phase II	27	NR	NR	NR	Treatment	17.2 (11.9-22.4)	16.9 (10.9-22.1)	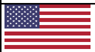
MMC + iodised oil + microspheres	Vogl [39]	2007	Prospective NRCS pilot study	12	0 (0%)	4 (33%)	NR	Treatment	21	21.0 (8.3-26.0)	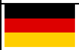
**Isolated hepatic perfusion (IHP)**											
Melphalan	Alexander [40]	2003	Prospective trial phase I/II	29	22 (76%)	0 (0%)	NR	Enrollment	12.1	12.5 (6.0-ND)	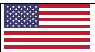
Melphalan w/wo TNF-alpha or cisplatin	Ben-Shabat [41]	2016	Retrospective case series	61	Majority	A few	NR	Treatment	22.4‡	23.9 (19.7- 26.0)	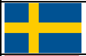
Melphalan	de Leede [42]	2016	Retrospective case series	31	27 (87%)	0 (0%)	2.3 (0.9-13.3)	Treatment	10	10.0 (6.9-13.9)	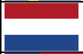
Melphalan	Forster [43]	2014	Retrospective case series	5	4 (80%)	0 (0%)	NR	Treatment	NR	14.2 (10.0-ND)	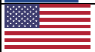
Oxaliplatin + melphalan	van Iersel [44]	2014	Prospective trial phase I	3	2 (67%)	0 (0%)	NR	Treatment	NR	18.7 (7.8-ND)	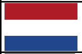
Melphalan	Vogl [45]	2017	Retrospective case series	18	7 (39)	5 (28)	NR	Treatment	9.6§	8.9 (4.4-23.5)	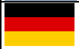
**Checkpoint inhibitor (CPI)**											
Pembrolizumab or nivolumab or atezolizumab	Algazi [46]	2016	Retrospective case series	56	8 (14%)	0 (0%)	NR	Treatment	7.7 (0.7-14.6)	7.6 (4.9-14.9)	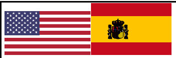
Ipilimumab	Danielli [47]	2012	Prospective NRCS	13	0 (0%)	NR	NR	Treatment	8.3	8.2 (3.7-15.9)	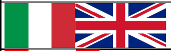
Tremelimumab	Joshua [48]	2015	Prospective trial phase II	11	NR**	3 (27%)	NR	Enrollment	12.8 (3.8-19.7)	12.8 (3.8-19.7)	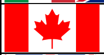
Pembrolizumab	Karydis [49]	2016	Retrospective case series	25	0 (0%)	3 (12%)	11.3 (3.7-65.1)	Treatment	NR	9.5 (5.0-14.1)	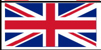
Ipilimumab	Kelderman [50]	2013	Retrospective case series, WIN-O	22	0 (0%)	NR	NR	Treatment	5.2††	4.6 (2.3-10.8)	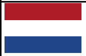
Ipilimumab	Luke [51]	2013	Retrospective case series	39	0 (0%)	NR	NR	Treatment	9.6 (6.3-13.4)	9.7 (6.4-13.4)	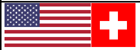

[*] In addition, 49 patients received intravenous fotemustine three weeks after TACE.

[†] In the Kaplan-Meier plot, some censored cases appear to be misaligned relative to the adjacent step and causing difficulty in digitizing.

[‡] The median differs either because of difficulty in digitizing the thick line with small steps in the published plot or because another method may have been used to calculate the median.

[§] A possible cause for the difference might be the dedicated statistical software used, because a very small difference in rounding would have shifted the step defining 0.5000 survival.

[**] Three patients had undergone surgery, three palliative radiation therapy, one TACE, three systemic chemotherapy.

[††] The reported median survival of 5.2 months appears to be arithmetically calculated and not a Kaplan-Meier estimate.

Ipilimumab	Maio [52]	2013	Prospective NRCS	83[*]	0 (0%)	NR	NR	NR	6.0 (4.3-7.7)	6.0 (4.6-8.4)	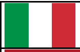
Nivolumab or pembrolizumab	van der Kooij [53]	2017	Retrospective case series	17	8 (47%)	NR	NR	Treatment	8.8	8.7 (3.7-ND)	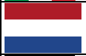
Ipilimumab	Zimmer [54]	2015	Prospective trial phase II	53	8 (15%)	NR	NR	Treatment	6.8 (3.7-8.1)	6.8 (3.7-8.2)	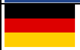
**Protein kinase inhibitor (PKI)**											
Sorafenib + carboplatin + paclitaxel	Bhatia [55]	2012	Prospective trial phase II	24	20 (83%)	NR	NR	Enrollment	11 (7-14)	10.5 (6.6-14.5)	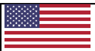
Cabozantinib	Daud [56]	2017	Prospective randomised discontinuation trial	23	NR[†]	NR	NR	Treatment	12.6	12.6 (4.6-19.9)	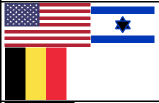
Imatinib	Hofmann [57]	2009	Prospective NRCS	12	8 (67%)	NR	NR	Treatment	6.8[‡]	5.5 (2.6-8.7)	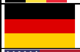
Sunitinib	Mahipal [58]	2012	Prospective NRCS pilot study	20	3 (15%)	3 (15%)	NR	Treatment	8.2	8.0 (4.6-14.2)	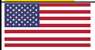
Sorafenib	Mouriaux [59]	2016	Prospective trial phase II	32	19 (59%)	0 (0%)	NR	Treatment	NR	7.8 (4.3-11.4)	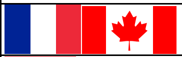
Sorafenib + fotemustine	Niederkorn[60]	2014	Retrospective case series	8	2 (25%)	NR	NR	Treatment	15.9	15.9 (5.6-20.3)	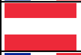
Imatinib mesylate	Penel[61]	2008	Prospective trial phase II	13	6 (46%)	0 (0%)	NR	Treatment	10.8	10.8 (1.2-ND)	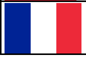
**Selective internal radiation therapy (SIRT)**											
SIRT, ^{90}Y resin microspheres	Eldredge-Hindy [62]	2016	Retrospective case series	50	13 (18%)[§]	NR	9.8	Treatment	NR	14.9 (9.7-17.2)	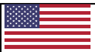
SIRT, ^{90}Y resin microspheres	Klingenstein [63]	2013	Retrospective case series	13	2 (15%)	1 (8%)	5 (1-49)	Treatment	7	7.0 (2.0-15.0)	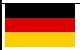
SIRT, ^{90}Y glass microspheres	Schelhorn [64]	2015	Retrospective case series	8	0 (0%)	0 (0%)	17.1 (6.4-23.2)	Treatment	2.8	2.8 (0.8-13.0)	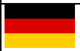
**Immunoembolisation (IE)**											
Immunoembolisation with GM-CSF	Sato [65]	2008	Prospective trial phase I	34	28 (82%)	3 (9%)	NR	Treatment	14.4[**]	18.4 (11.2-22.8)	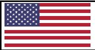
Immunoembolisation with GM-CSF	Valsecchi [38]	2015	Prospective trial phase II	25	NR	NR	NR	Treatment	21.5 (18.5-24.8)	21.6 (17.5-30.1)	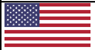
**Immunosuppressant (IS)**											
Everolimus + pasireotide	Shoushtari [66]	2016	Prospective trial phase II	14	0 (0%)	NR	NR	Treatment	11	10.9 (5.4-17.6)	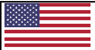

[*] One patient was lost in follow-up of 83 patients and in Kaplan-Meier plot 82 patients are depicted.

[†] Reported for all patients, not specifically for uveal melanoma patients.

[‡] The reported median survival of 6.8 months appears to correspond to mean survival [(1.59+2.62+3.60+4.57+4.57+5.55+5.67+6.59+6.65+8.66+10.73+ 20.73)/12=6.8 months, from the digitized Kaplan-Meier plot].

[§] Prior treatment reported for all 58 patients, and not specifically for the 50 patients who had a pretreatment positron emission tomography/computer tomography and were included in the Kaplan-Meier plot.

[**] The median overall survival is reported for 34 patients in intent-to-treat analysis whereas the Kaplan-Meier plot incudes 31 radiographically assessable patients.

**Liver-directed thermotherapy (LDT)**											
Stereotactic radiofrequency ablation	Bale [67]	2016	Retrospective case series	6	0 (0%)	0 (0%)	NR	Treatment	38[*]	36.2 (15.7-ND)	
Laser-induced thermotherapy	Eichler [68]	2014	Retrospective case series	18	NR[†]	NR	4 (22)	Diagnosis	29.2[‡]	29.2 (12.5-43.0)	
**Vaccine**											
Dendritic cell vaccine	Bol [69]	2014	Prospective NRCS	14	8 (57%)	3 (21%)	NR	Treatment	19.2	19.4 (6.8-38.1)	
**Surgery**											
Resection	Aoyama [70]	2000	Retrospective case series	12	8 (67%)	0 (0%)	NR	Treatment	NR	27 (23-ND)	
Resection[§]<br>R0<br>R1/R2	Frenkel [71]	2009	Retrospective CCS	 14<br>23	 NR<br>NR	 0 (0%)<br>0 (0%)	 NR<br>NR	 Diagnosis<br>Diagnosis	 NR<br>NR	 65.2 (11.2-ND)<br>16.3 (7.6-25.3)	
Resection[**]	Gomez [72]	2014	Retrospective CCS	18	NR	NR	NR	Treatment	27[††]	28.9 (20.0-ND)	
Resection	Hsueh [73]	2004	Retrospectice CCS	24	NR[‡‡]	NR	NR	Diagnosis	38[††]	35.7 (21.2-ND)	
Resection + fotemustine IA or cisplatin IA<br><br>R0<br>R2[§§]	Kodjikian [74] / Rivoire [75]	2005	Retrospective CCS	 <br>14<br>14	 <br>14 (100%)<br>14 (100%)	 <br>0 (0%)<br>0 (0%)	 <br>NR<br>NR	 <br>Treatment<br>Treatment	 <br>25[***]<br>16	 <br>35.3 (15.0-ND)<br>16.3 (11.0-17.6)	
 Resection<br>Resection + RFA	Mariani [76]	2016	Retrospective CCS	 57<br>13	 57 (100%)<br>13 (100%)	 0 (0%)<br>0 (0%)	 NR<br>NR	 Treatment<br>Treatment	 27<br>28	 26.9 (20.8-34.8)<br>27.8 (16.9-ND)	

[*] In the original publication median survival was reported as 38 months; according to personal communication this was calculated by the actuarial method and is 36.3 months by the Kaplan-Meier method.

[†] The authors state: “Limitations of our study are the small number of patients and the inhomogenous population concerning various treatments prior the LITT like immunochemotherapy and TACE”.

[‡] Personal communication.

[§]The authors state: “Some patients received chemotherapy intravenously or through an intra-arterial hepatic port, and some received adjuvant immunotherapy”.

[**] One RFA.

[††]The reported median survival appears to be arithmetically calculated and not a Kaplan-Meier estimate.

[‡‡] Of 112 treatments, 13 were first-line, but the numbers are not reported separately for the 24 patients who underwent surgery; in addition, some resections were combined with immunotherapy, systemic chemotherapy, and hepatic perfusion.

[§§] Three patients received no chemotherapy.

[***] The reported median survival appears to be arithmetically calculated and not a Kaplan-Meier estimate.

Resection*<br>R0<br>R1<br>R2	Mariani [77]	2009	Retrospective comparative case series	 76<br>22<br>157	 76 (100%)<br>22 (100%)<br>157 (100%)	 0 (0%)<br>0 (0%)<br>0 (0%)	 NR<br>NR<br>NR	 Treatment<br>Treatment<br>Treatment	 27<br>17†<br>11	 27.0 (18.2-32.0)<br>16.0 (7.1-30.1)<br>11.1 (9.0-12.0)	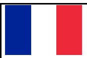
Resection + fotemustine IA or dacarbazine IA + cisplatin IA<br>Curative resection<br>Tumour reduction	Salmon [23]	1998	Prospective nonrandomised comparative case series	 19<br>34	 19 (100%)<br>34 (100%)	 0 (0%)<br>0 (0%)	 NR<br>NR	 NR<br>NR	 22<br>NR	 30.1 (17.1-ND)‡<br>8.1 (5.3-10.1)	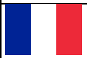
Resection, partial§	Yang [78]	2013	Retrospective case series	5	NR	NR	NR	Treatment	11.5 (7.5-15.8)	11.5 (8.5-ND)	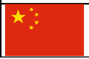

Abbreviations

BCNU, bis-chloroethylnitrosourea; BOLD, includes bleomycin, vincristine, lomustine, and dacarbazine; CCS, comparative case series; CHT, conventional chemotherapy; CI, confidence interval; CIT, chemoimmunotherapy; CPI, checkpoint inhibitor; GM-CSF, granulocyte-macrophage colony-stimulating factor; HIA, hepatic intra-arterial chemotherapy; IA, intra-arterial; IFN, interferon; IHP, isolated hepatic perfusion; IL2, interleukin 2; IV, intravenous; LDT, liver-directed thermotherapy; LITT, laser-induced thermotherapy; MMC, mitomycin; NC, no prior chemotherapy nor systemic treatment; ND, not defined; NR, not reported; NRCS, nonrandomised controlled case series; OS, overall survival; PKI, protein kinase inhibitor; R0, microscopically complete liver surgery; R1, microscopically incomplete liver surgery; R2, macroscopically incomplete liver surgery; RFA, radiofrequency ablation; SIRT, selective internal radiation therapy; TACE, transarterial chemoembolisation; TNF, tumour necrosis factor.

* The authors state: "Postoperative chemotherapy was mandated in every patient during the first years of our experience, but since 2005 has been reserved for randomised patients in the intra-arterial arm of the European trial"; in addition, eight patients underwent RFA during laparotomy to attain R0 resection.

† A possible cause for the difference might be the dedicated statistical software used, because a very small difference in rounding of decimals would have shifted the step defining 0.5000 survival.

‡ A possible cause for the difference might be the dedicated statistical software used, because a very small difference in rounding of decimals would have shifted the step defining 0.5000 survival; the adjacent steps in the plot are 22.2 and 30.1 months, and from the Kaplan-Meier plot it is clear that the median survival indeed is about 22 months, and 22.2 was used in our analysis.

§ Of the five patients, two received adjuvant therapy with TACE with or without immunotherapy.

References

1 Carling U, Dorenberg EJ, Haugvik SP, Eide NA, Berntzen DT, Edwin B, *et al.* Transarterial chemoembolization of liver metastases from uveal melanoma using irinotecan-loaded beads: treatment response and complications. *Cardiovasc Intervent Radiol* 2015; **38**:1532-1541.

2 Corrie PG, Shaw J, Spanswick VJ, Sehmbi R, Jonson A, Mayer A, *et al*. Phase I trial combining gemcitabine and treosulfan in advanced cutaneous and uveal melanoma patients. *Br J Cancer* 2005; **92**:1997-2003.

3 Homsi J, Bedikian AY, Papadopoulos NE, Kim KB, Hwu WJ, Mahoney SL, et al. Phase 2 open-label study of weekly docosahexaenoic acid-paclitaxel in patients with metastatic uveal melanoma. *Melanoma Res* 2010; **20**:507-510.

4 Leyvraz S, Piperno-Neumann S, Suciu S, Baurain JF, Zdzienicki M, Testori A, *et al.* Hepatic intra-arterial versus intravenous fotemustine in patients with liver metastases from uveal melanoma (EORTC 18021): a multicentric randomized trial. *Ann Oncol* 2014; **25**:742-746.

5 Pföhler C, Cree IA, Ugurel S, Kuwert C, Haass N, Neuber K, et al. Treosulfan and gemcitabine in metastatic uveal melanoma patients: results of a multicenter feasibility study. *Anticancer Drugs* 2003; **14**:337-340.

6 Piperno-Neumann S, Diallo A, Etienne-Grimaldi MC, Bidard FC, Rodrigues M, Plancher C, *et al.* Phase II trial of bevacizumab in combination with temozolomide as first-line treatment in patients with metastatic uveal melanoma. *Oncologist* 2016; **21**:281-282.

7 Pons F, Plana M, Caminal JM, Pera J, Fernandes I, Perez J, *et al*. Metastatic uveal melanoma: is there a role for conventional chemotherapy? - A single center study based on 58 patients. *Melanoma Res* 2011; **21**:217-222.

8 Schmittel A, Scheulen ME, Bechrakis NE, Strumberg D, Baumgart J, Bornfeld N, *et al.* Phase II trial of cisplatin, gemcitabine and treosulfan in patients with metastatic uveal melanoma. *Melanoma Res* 2005; **15**:205-207.

9 Schmittel A, Schuster R, Bechrakis NE, Siehl JM, Foerster MH, Thiel E, *et al.* A two-cohort phase II clinical trial of gemcitabine plus treosulfan in patients with metastatic uveal melanoma. *Melanoma Res* 2005; **15**:447-451.

10 Terheyden P, Bröcker EB, Becker JC. Clinical evaluation of in vitro chemosensitivity testing: the example of uveal melanoma. *J Cancer Res Clin Oncol* 2004; **130**:395-399.

11 Becker JC, Terheyden P, Kämpgen E, Wagner S, Neumann C, Schadendorf D, *et al*. Treatment of disseminated ocular melanoma with sequential fotemustine, interferon alpha, and interleukin 2. *Br J Cancer* 2002; **87**:840-845.

12 Kivelä T, Suciu S, Hansson J, Kruit WH, Vuoristo MS, Kloke O, *et al.* Bleomycin, vincristine, lomustine and dacarbazine (BOLD) in combination with recombinant interferon alpha-2b for metastatic uveal melanoma. *Eur J Cancer* 2003; **39**:1115-1120.

13 Pyrhönen S, Hahka-Kemppinen M, Muhonen T, Nikkanen V, Eskelin S, Summanen P, *et al.* Chemoimmunotherapy with bleomycin, vincristine, lomustine, dacarbazine (BOLD), and human leukocyte interferon for metastatic uveal melanoma. *Cancer* 2002; **95**:2366-2372.

14 Solti M, Berd D, Mastrangelo MJ, Sato T. A pilot study of low-dose thalidomide and interferon alpha-2b in patients with metastatic melanoma who failed prior treatment. *Melanoma Res* 2007; **17**:225-231.

15 Terheyden P, Kämpgen E, Rünger TM, Bröcker EB, Becker JC. Immunochemotherapie des metastasierenden Uveamelanoms mit Interferon-alpha 2b, Interleukin-2 und Fotemustin. Kasuistiken und Literaturübersicht. *Hautarzt* 1998; **49**:770-773.

16 Vihinen PP, Hernberg M, Vuoristo MS, Tyynelä K, Laukka M, Lundin J, *et al.* A phase II trial of bevacizumab with dacarbazine and daily low-dose interferon-alpha2a as first line treatment in metastatic melanoma. *Melanoma Res* 2010; **20**:318-325.

17 Cantore M, Fiorentini G, Aitini E, Davitti B, Cavazzini G, Rabbi C, *et al.* Intra-arterial hepatic carboplatin-based chemotherapy for ocular melanoma metastatic to the liver. Report of a phase II study. *Tumori* 1994; **80**:37-39.

18 Egerer G, Lehnert T, Max R, Naeher H, Keilholz U, Ho AD. Pilot study of hepatic intraarterial fotemustine chemotherapy for liver metastases from uveal melanoma: a single-center experience with seven patients. *Int J Clin Oncol* 2001; **6**:25-28.

19 Farolfi A, Ridolfi L, Guidoboni M, Milandri C, Calzolari F, Scarpi E, *et al.* Liver metastases from melanoma: hepatic intra-arterial chemotherapy. A retrospective study. *J Chemother* 2011; **23**:300-305.

20 Heusner TA, Antoch G, Wittkowski-Sterczewski A, Ladd SC, Forsting M, Verhagen R, *et al.* Transarterial hepatic chemoperfusion of uveal melanoma metastases: survival and response to treatment. *Rofo* 2011; **183**:1151-1160.

21 Leyvraz S, Spataro V, Bauer J, Pampallona S, Salmon R, Dorval T, *et al*. Treatment of ocular melanoma metastatic to the liver by hepatic arterial chemotherapy. *J Clin Oncol* 1997; **15**:2589-2595.

22 Melichar B, Vobořil Z, Lojik M, Krajina A. Liver metastases from uveal melanoma: clinical experience of hepatic arterial infusion of cisplatin, vinblastine and dacarbazine. *Hepatogastroenterology* 2009; **56**:1157-1162.

23 Salmon RJ, Levy C, Plancher C, Dorval T, Desjardins L, Leyvraz S, *et al.* Treatment of liver metastases from uveal melanoma by combined surgery-chemotherapy. *Eur J Surg Oncol* 1998; **24**:127-130.

24 Siegel R, Hauschild A, Kettelhack C, Kähler KC, Bembenek A, Schlag PM. Hepatic arterial fotemustine chemotherapy in patients with liver metastases from cutaneous melanoma is as effective as in ocular melanoma. *Eur J Surg Oncol* 2007; **33**:627-632.
25 Peters S, Voelter V, Zografos L, Pampallona S, Popescu R, Gillet M, *et al.* Intra-arterial hepatic fotemustine for the treatment of liver metastases from uveal melanoma: experience in 101 patients. *Ann Oncol* 2006; **17**:578-583.
26 Agarwala SS, Panikkar R, Kirkwood JM. Phase I/II randomized trial of intrahepatic arterial infusion chemotherapy with cisplatin and chemoembolization with cisplatin and polyvinyl sponge in patients with ocular melanoma metastatic to the liver. *Melanoma Res* 2004; **14**:217-222.
27 Dayani PN, Gould JE, Brown DB, Sharma KV, Linette GP, Harbour JW. Hepatic metastasis from uveal melanoma: angiographic pattern predictive of survival after hepatic arterial chemoembolization. *Arch Ophthalmol* 2009; **127**:628-632.
28 Edelhauser G, Schicher N, Berzaczy D, Beitzke D, Höeller C, Lammer J, *et al.* Fotemustine chemoembolization of hepatic metastases from uveal melanoma: a retrospective single-center analysis. *American journal of roentgenology* 2012; **199**:1387-1392.
29 Fiorentini G, Aliberti C, Del Conte A, Tilli M, Rossi S, Ballardini P, *et al.* Intra-arterial hepatic chemoembolization (TACE) of liver metastases from ocular melanoma with slow-release irinotecan-eluting beads. Early results of a phase II clinical study. *In Vivo* 2009; **23**:131-137.
30 Gonsalves CF, Eschelman DJ, Thornburg B, Frangos A, Sato T. Uveal melanoma metastatic to the liver: chemoembolization with 1,3-bis-(2-chloroethyl)-1-nitrosourea. *American journal of roentgenology* 2015; **205**:429-433.
31 Gupta S, Bedikian AY, Ahrar J, Ensor J, Ahrar K, Madoff DC, *et al.* Hepatic artery chemoembolization in patients with ocular melanoma metastatic to the liver: response, survival, and prognostic factors. *Am J Clin Oncol* 2010; **33**:474-480.
32 Huppert PE, Fierlbeck G, Pereira P, Schanz S, Duda SH, Wietholtz H, *et al.* Transarterial chemoembolization of liver metastases in patients with uveal melanoma. *Eur J Radiol* 2010; **74**:e38-44.
33 Mavligit GM, Charnsangavej C, Carrasco CH, Patt YZ, Benjamin RS, Wallace S. Regression of ocular melanoma metastatic to the liver after hepatic arterial chemoembolization with cisplatin and polyvinyl sponge. *JAMA* 1988; **260**:974-976.
34 Patel K, Sullivan K, Berd D, Mastrangelo MJ, Shields CL, Shields JA, *et al.* Chemoembolization of the hepatic artery with BCNU for metastatic uveal melanoma: results of a phase II study. *Melanoma Res* 2005; **15**:297-304.
35 Schuster R, Lindner M, Wacker F, Krössin M, Bechrakis N, Foerster MH, *et al.* Transarterial chemoembolization of liver metastases from uveal melanoma after failure of systemic therapy: toxicity and outcome. *Melanoma Res* 2010; **20**:191-196.
36 Shibayama Y, Namikawa K, Sone M, Takahashi A, Tsutsumida A, Sugawara S, *et al.* Efficacy and toxicity of transarterial chemoembolization therapy using cisplatin and gelatin sponge in patients with liver metastases from uveal melanoma in an Asian population. *Int J Clin Oncol* 2017.
37 Valpione S, Aliberti C, Parrozzani R, Bazzi M, Pigozzo J, Midena E, *et al.* A retrospective analysis of 141 patients with liver metastases from uveal melanoma: a two-cohort study comparing transarterial chemoembolization with CPT-11 charged microbeads and historical treatments. *Melanoma Res* 2015; **25**:164-168.
38 Valsecchi ME, Terai M, Eschelman DJ, Gonsalves CF, Chervoneva I, Shields JA, *et al.* Double-blinded, randomized phase II study using embolization with or without granulocyte-macrophage colony-stimulating factor in uveal melanoma with hepatic metastases. *J Vasc Interv Radiol* 2015; **26**:523-532.e522.
39 Vogl T, Eichler K, Zangos S, Herzog C, Hammerstingl R, Balzer J, *et al.* Preliminary experience with transarterial chemoembolization (TACE) in liver metastases of uveal malignant melanoma: local tumor control and survival. *J Cancer Res Clin Oncol* 2007; **133**:177-184.
40 Alexander HR, Jr., Libutti SK, Pingpank JF, Steinberg SM, Bartlett DL, Helsabeck C, *et al.* Hyperthermic isolated hepatic perfusion using melphalan for patients with ocular melanoma metastatic to liver. *Clin Cancer Res* 2003; **9**:6343-6349.
41 Ben-Shabat I, Belgrano V, Ny L, Nilsson J, Lindnér P, Olofsson Bagge R. Long-term follow-up evaluation of 68 patients with uveal melanoma liver metastases treated with isolated hepatic perfusion. *Ann Surg Oncol* 2016; **23**:1327-1334.
42 de Leede EM, Burgmans MC, Kapiteijn E, Luyten GP, Jager MJ, Tijl FG, *et al.* Isolated (hypoxic) hepatic perfusion with high-dose chemotherapy in patients with unresectable liver metastases of uveal melanoma: results from two experienced centres. *Melanoma Res* 2016; **26**:588-594.
43 Forster MR, Rashid OM, Perez MC, Choi J, Chaudhry T, Zager JS. Chemosaturation with percutaneous hepatic perfusion for unresectable metastatic melanoma or sarcoma to the liver: a single institution experience. *J Surg Oncol* 2014; **109**:434-439.
44 van Iersel LB, de Leede EM, Vahrmeijer AL, Tijl FG, den Hartigh J, Kuppen PJ, *et al.* Isolated hepatic perfusion with oxaliplatin combined with 100 mg melphalan in patients with metastases confined to the liver: A phase I study. *Eur J Surg Oncol* 2014; **40**:1557-1563.

45 Vogl TJ, Koch SA, Lotz G, Gebauer B, Willinek W, Engelke C, *et al.* Percutaneous isolated hepatic perfusion as a treatment for isolated hepatic metastases of uveal melanoma: patient outcome and safety in a multi-centre study. *Cardiovasc Intervent Radiol* 2017.

46 Algazi AP, Tsai KK, Shoushtari AN, Munhoz RR, Eroglu Z, Piulats JM, *et al*. Clinical outcomes in metastatic uveal melanoma treated with PD-1 and PD-L1 antibodies. *Cancer* 2016; **122**:3344-3353.

47 Danielli R, Ridolfi R, Chiarion-Sileni V, Queirolo P, Testori A, Plummer R, *et al*. Ipilimumab in pretreated patients with metastatic uveal melanoma: safety and clinical efficacy. *Cancer Immunol Immunother* 2012; **61**:41-48.

48 Joshua AM, Monzon JG, Mihalcioiu C, Hogg D, Smylie M, Cheng T. A phase 2 study of tremelimumab in patients with advanced uveal melanoma. *Melanoma Res* 2015; **25**:342-347.

49 Karydis I, Chan PY, Wheater M, Arriola E, Szlosarek PW, Ottensmeier CH. Clinical activity and safety of pembrolizumab in ipilimumab pre-treated patients with uveal melanoma. *Oncoimmunology* 2016; **5**:e1143997.

50 Kelderman S, van der Kooij MK, van den Eertwegh AJ, Soetekouw PM, Jansen RL, van den Brom RR, *et al*. Ipilimumab in pretreated metastastic uveal melanoma patients. Results of the Dutch Working Group on Immunotherapy of Oncology (WIN-O). *Acta Oncol* 2013; **52**:1786-1788.

51 Luke JJ, Callahan MK, Postow MA, Romano E, Ramaiya N, Bluth M, *et al*. Clinical activity of ipilimumab for metastatic uveal melanoma: a retrospective review of the Dana-Farber Cancer Institute, Massachusetts General Hospital, Memorial Sloan-Kettering Cancer Center, and University Hospital of Lausanne experience. *Cancer* 2013; **119**:3687-3695.

52 Maio M, Danielli R, Chiarion-Sileni V, Pigozzo J, Parmiani G, Ridolfi R, *et al*. Efficacy and safety of ipilimumab in patients with pre-treated, uveal melanoma. *Ann Oncol* 2013; **24**:2911-2915.

53 van der Kooij MK, Joosse A, Speetjens FM, Hospers GA, Bisschop C, de Groot JW, *et al*. Anti-PD1 treatment in metastatic uveal melanoma in the Netherlands. *Acta Oncol* 2017; **56**:101-103.

54 Zimmer L, Vaubel J, Mohr P, Hauschild A, Utikal J, Simon J, *et al*. Phase II DeCOG-study of ipilimumab in pretreated and treatment-naive patients with metastatic uveal melanoma. *PLoS One* 2015; **10**:e0118564.

55 Bhatia S, Moon J, Margolin KA, Weber JS, Lao CD, Othus M, *et al*. Phase II trial of sorafenib in combination with carboplatin and paclitaxel in patients with metastatic uveal melanoma: SWOG S0512. *PLoS One* 2012; **7**:e48787.

56 Daud A, Kluger HM, Kurzrock R, Schimmoller F, Weitzman AL, Samuel TA, *et al*. Phase II randomised discontinuation trial of the MET/VEGF receptor inhibitor cabozantinib in metastatic melanoma. *Br J Cancer* 2017; **116**:432-440.

57 Hofmann UB, Kauczok-Vetter CS, Houben R, Becker JC. Overexpression of the KIT/SCF in uveal melanoma does not translate into clinical efficacy of imatinib mesylate. *Clin Cancer Res* 2009; **15**:324-329.

58 Mahipal A, Tijani L, Chan K, Laudadio M, Mastrangelo MJ, Sato T. A pilot study of sunitinib malate in patients with metastatic uveal melanoma. *Melanoma Res* 2012; **22**:440-446.

59 Mouriaux F, Servois V, Parienti JJ, Lesimple T, Thyss A, Dutriaux C, *et al*. Sorafenib in metastatic uveal melanoma: efficacy, toxicity and health-related quality of life in a multicentre phase II study. *Br J Cancer* 2016; **115**:20-24.

60 Niederkorn A, Wackernagel W, Artl M, Schwantzer G, Aigner B, Richtig E. Response of patients with metastatic uveal melanoma to combined treatment with fotemustine and sorafenib. *Acta Ophthalmol* 2014; **92**:e696-697.

61 Penel N, Delcambre C, Durando X, Clisant S, Hebbar M, Negrier S, *et al*. O-Mel-Inib: a Cancero-pole Nord-Ouest multicenter phase II trial of high-dose imatinib mesylate in metastatic uveal melanoma. *Invest New Drugs* 2008; **26**:561-565.

62 Eldredge-Hindy H, Ohri N, Anne PR, Eschelman D, Gonsalves C, Intenzo C, *et al*. Yttrium-90 microsphere brachytherapy for liver metastases from uveal melanoma: clinical outcomes and the predictive value of fluorodeoxyglucose positron emission tomography. *Am J Clin Oncol* 2016; **39**:189-195.

63 Klingenstein A, Haug AR, Zech CJ, Schaller UC. Radioembolization as locoregional therapy of hepatic metastases in uveal melanoma patients. *Cardiovasc Intervent Radiol* 2013; **36**:158-165.

64 Schelhorn J, Richly H, Ruhlmann M, Lauenstein TC, Theysohn JM. A single-center experience in radioembolization as salvage therapy of hepatic metastases of uveal melanoma. *Acta Radiol Open* 2015; **4**:2047981615570417.

65 Sato T, Eschelman DJ, Gonsalves CF, Terai M, Chervoneva I, McCue PA, *et al*. Immunoembolization of malignant liver tumors, including uveal melanoma, using granulocyte-macrophage colony-stimulating factor. *J Clin Oncol* 2008; **26**:5436-5442.

66 Shoushtari AN, Ong LT, Schoder H, Singh-Kandah S, Abbate KT, Postow MA, *et al*. A phase 2 trial of everolimus and pasireotide long-acting release in patients with metastatic uveal melanoma. *Melanoma Res* 2016; **26**:272-277.

67 Bale R, Schullian P, Schmuth M, Widmann G, Jaschke W, Weinlich G. Stereotactic radiofrequency ablation for metastatic melanoma to the liver. *Cardiovasc Intervent Radiol* 2016; **39**:1128-1135.

68 Eichler K, Zangos S, Gruber-Rouh T, Vogl TJ, Mack MG. MR-guided laser-induced thermotherapy (LITT) in patients with liver metastases of uveal melanoma. *J Eur Acad Dermatol Venereol* 2014; **28**:1756-1760.
69 Bol KF, Mensink HW, Aarntzen EH, Schreibelt G, Keunen JE, Coulie PG, *et al*. Long overall survival after dendritic cell vaccination in metastatic uveal melanoma patients. *Am J Ophthalmol* 2014; **158**:939-947.
70 Aoyama T, Mastrangelo MJ, Berd D, Nathan FE, Shields CL, Shields JA, *et al*. Protracted survival after resection of metastatic uveal melanoma. *Cancer* 2000; **89**:1561-1568.
71 Frenkel S, Nir I, Hendler K, Lotem M, Eid A, Jurim O, *et al*. Long-term survival of uveal melanoma patients after surgery for liver metastases. *Br J Ophthalmol* 2009; **93**:1042-1046.
72 Gomez D, Wetherill C, Cheong J, Jones L, Marshall E, Damato B, *et al*. The Liverpool uveal melanoma liver metastases pathway: outcome following liver resection. *J Surg Oncol* 2014; **109**:542-547.
73 Hsueh EC, Essner R, Foshag LJ, Ye X, Wang HJ, Morton DL. Prolonged survival after complete resection of metastases from intraocular melanoma. *Cancer* 2004; **100**:122-129.
74 Kodjikian L, Grange JD, Baldo S, Baillif S, Garweg JG, Rivoire M. Prolonged survival after resection of liver metastases from uveal melanoma and intra-arterial chemotherapy. *Graefes Arch Clin Exp Ophthalmol* 2005; **243**:622-624.
75 Rivoire M, Kodjikian L, Baldo S, Kaemmerlen P, Négrier S, Grange JD. Treatment of liver metastases from uveal melanoma. *Ann Surg Oncol* 2005; **12**:422-428.
76 Mariani P, Almubarak MM, Kollen M, Wagner M, Plancher C, Audollent R, *et al*. Radiofrequency ablation and surgical resection of liver metastases from uveal melanoma. *Eur J Surg Oncol* 2016; **42**:706-712.
77 Mariani P, Piperno-Neumann S, Servois V, Berry MG, Dorval T, Plancher C, *et al*. Surgical management of liver metastases from uveal melanoma: 16 years' experience at the Institut Curie. *Eur J Surg Oncol* 2009; **35**:1192-1197.
78 Yang XY, Xie F, Tao R, Li AJ, Wu MC. Treatment of liver metastases from uveal melanoma: a retrospective single-center analysis. *Hepatobiliary Pancreat Dis Int* 2013; **12**:602-606.
