# Supplementary material for: Overall survival after treatment for metastatic uveal melanoma: a systematic review and meta-analysis
Source: Melanoma Res. 2019 Jan 16;29(6):561–8. doi: 10.1097/CMR.0000000000000575 (PMC6887637; doi:10.1097/CMR.0000000000000575)
Supplement: Supplementary file 3 [file mr-29-561-s003.pdf]

Supplemental Digital Content 3 – Excluded studies

We excluded six articles because of uncertainty whether they shared patients with other publications: Alexander *et al*. 2000 [1] and Varghese *et al*. 2010 [2] (excluded) with Alexander *et al*. 2003 [3]; Ben-Shabat *et al*. 2017 [4] and Olofsson *et al*. 2014 [5] (excluded) with Ben-Shabat *et al*. 2016 [6]; Gonsalves *et al*. 2011 [7] (excluded) with Eldredge-Hindy *et al*. 2014 [8]; and Noter *et al*. 2004 [9] (excluded) with de Leede *et al*. 2016 [10]. We tabulate Kodjikian *et al*. 2005 [11] and Rivoire *et al*. 2005 [12] together as they apparently overlap completely.

In one article, a "nonsurgery" subgroup of 86 patients could not be assigned to specific modality [13]. A subgroup with combination chemotherapies in the article by Pons et al. was included in the pooled conventional chemotherapy (CHT) group but could not be analysed by agent, and six surgically treated patients did not have individual survival times [14].

References

1 Alexander HR, Libutti SK, Bartlett DL, Puhlmann M, Fraker DL, Bachenheimer LC. A phase I-II study of isolated hepatic perfusion using melphalan with or without tumor necrosis factor for patients with ocular melanoma metastatic to liver. *Clin Cancer Res* 2000; **6**:3062-3070.

2 Varghese S, Xu H, Bartlett D, Hughes M, Pingpank JF, Beresnev T, *et al*. Isolated hepatic perfusion with high-dose melphalan results in immediate alterations in tumor gene expression in patients with metastatic ocular melanoma. *Ann Surg Oncol* 2010; **17**:1870-1877.

3 Alexander HR, Jr., Libutti SK, Pingpank JF, Steinberg SM, Bartlett DL, Helsabeck C, *et al*. Hyperthermic isolated hepatic perfusion using melphalan for patients with ocular melanoma metastatic to liver. *Clin Cancer Res* 2003; **9**:6343-6349.

4 Ben-Shabat I, Belgrano V, Hansson C, Olofsson Bagge R. The effect of perfusate buffering on toxicity and response in isolated hepatic perfusion for uveal melanoma liver metastases. *Int J Hyperthermia* 2017:1-17.

5 Olofsson R, Cahlin C, All-Ericsson C, Hashimi F, Mattsson J, Rizell M, *et al*. Isolated hepatic perfusion for ocular melanoma metastasis: registry data suggests a survival benefit. *Ann Surg Oncol* 2014; **21**:466-472.

6 Ben-Shabat I, Belgrano V, Ny L, Nilsson J, Lindnér P, Olofsson Bagge R. Long-term follow-up evaluation of 68 patients with uveal melanoma liver metastases treated with isolated hepatic perfusion. *Ann Surg Oncol* 2016; **23**:1327-1334.

7 Gonsalves CF, Eschelman DJ, Sullivan KL, Anne PR, Doyle L, Sato T. Radioembolization as salvage therapy for hepatic metastasis of uveal melanoma: a single-institution experience. *American journal of roentgenology* 2011; **196**:468-473.

8 Eldredge-Hindy H, Ohri N, Anne PR, Eschelman D, Gonsalves C, Intenzo C, *et al*. Yttrium-90 microsphere brachytherapy for liver metastases from uveal melanoma: clinical outcomes and the predictive value of fluorodeoxyglucose positron emission tomography. *Am J Clin Oncol* 2016; **39**:189-195.

9 Noter SL, Rothbarth J, Pijl ME, Keunen JE, Hartgrink HH, Tijl FG, *et al*. Isolated hepatic perfusion with high-dose melphalan for the treatment of uveal melanoma metastases confined to the liver. *Melanoma Res* 2004; **14**:67-72.

10 de Leede EM, Burgmans MC, Kapiteijn E, Luyten GP, Jager MJ, Tijl FG, *et al*. Isolated (hypoxic) hepatic perfusion with high-dose chemotherapy in patients with unresectable liver metastases of uveal melanoma: results from two experienced centres. *Melanoma Res* 2016; **26**:588-594.

11 Kodjikian L, Grange JD, Baldo S, Baillif S, Garweg JG, Rivoire M. Prolonged survival after resection of liver metastases from uveal melanoma and intra-arterial chemotherapy. *Graefes Arch Clin Exp Ophthalmol* 2005; **243**:622-624.

12 Rivoire M, Kodjikian L, Baldo S, Kaemmerlen P, Négrier S, Grange JD. Treatment of liver metastases from uveal melanoma. *Ann Surg Oncol* 2005; **12**:422-428.

13 Hsueh EC, Essner R, Foshag LJ, Ye X, Wang HJ, Morton DL. Prolonged survival after complete resection of metastases from intraocular melanoma. *Cancer* 2004; **100**:122-129.

14 Pons F, Plana M, Caminal JM, Pera J, Fernandes I, Perez J, *et al*. Metastatic uveal melanoma: is there a role for conventional chemotherapy? **-** A single center study based on 58 patients. *Melanoma Res* 2011; **21**:217-222.
