# Supplementary material for: Overall survival after treatment for metastatic uveal melanoma: a systematic review and meta-analysis
Source: Melanoma Res. 2019 Jan 16;29(6):561–8. doi: 10.1097/CMR.0000000000000575 (PMC6887637; doi:10.1097/CMR.0000000000000575)
Supplement: Supplementary file 4 [file mr-29-561-s004.pdf]

Supplemental digital content 4.pdf - Supplemental figures

Fig. S1. Kaplan-Meier plots (A-M) of overall survival after metastatic uveal melanoma by treatment modality, digitised data for each individual study to display heterogeneity. The spread of the survival curves that derives from differences in study design, inclusion criteria, and case mix may be viewed as an empiric representation of a confidence interval for each modality. The percentage within brackets refers to first-line treatments. *P* values were calculated by log-rank test. Abbreviations: NR not reported.

A. Conventional chemotherapy (CHT)

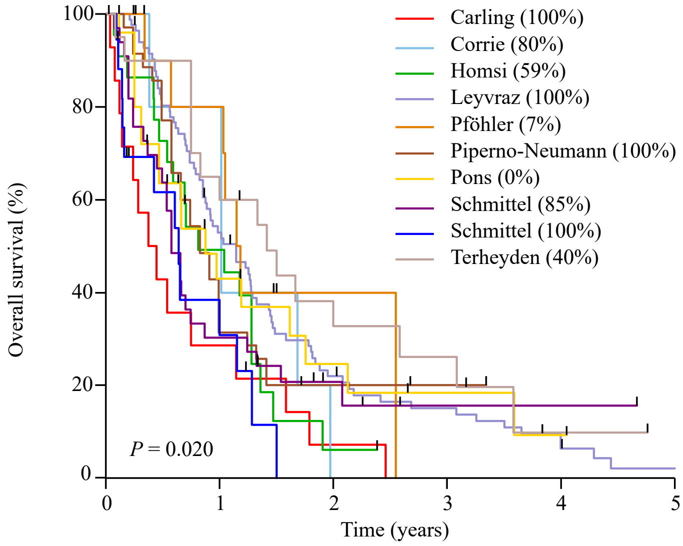

Number at risk

	0	1	2	3	4	5
Carling [1]	14	4	1	0	0	0
Corrie [2]	5	4	0	0	0	0
Homsi [3]	22	10	1	0	0	0
Leyvraz [4]	85	42	17	11	3	1
Pföhler [5]	14	8	1	0	0	0
Piperno-Neumann [6]	35	11	4	1	0	0
Pons [7]	25	8	4	2	1	0
Schmittel [9]	33	10	4	1	1	0
Schmittel [8]	19	4	0	0	0	0
Terheyden [10]	20	13	7	4	1	0

B. Chemoimmunotherapy (CIT)

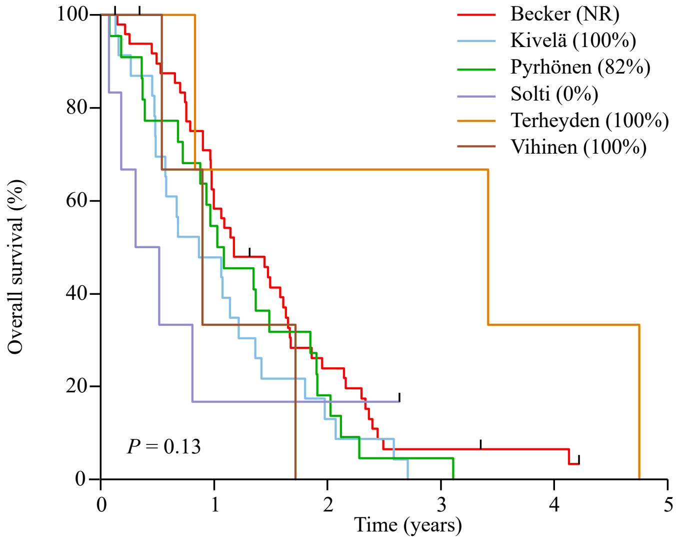

Number at risk

	0	1	2	3	4	5
Becker [11]	48	29	11	3	2	0
Kivelä [12]	24	11	3	0	0	0
Pyrhönen[13]	22	12	4	1	0	0
Solti [14]	6	1	1	0	0	0
Terheyden [15]	3	2	2	2	1	0
Vihinen [16]	4	1	0	0	0	0

C. Hepatic intra-arterial chemotherapy (HIA)

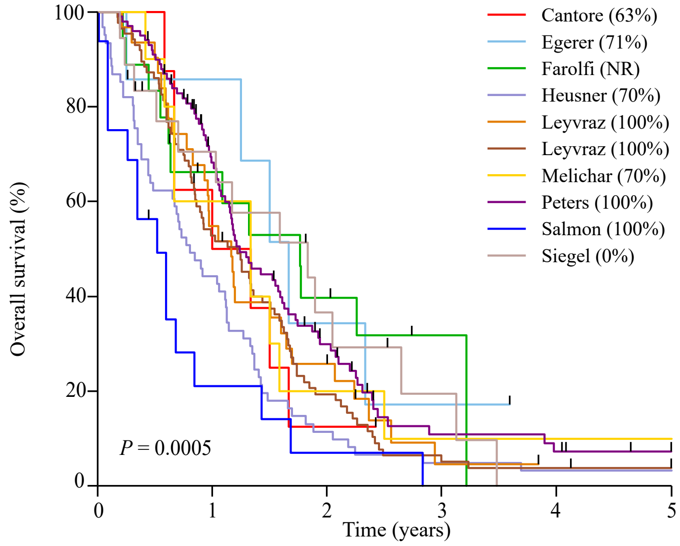

Number at risk

	0	1	2	3	4	5
Cantore [17]	8	5	1	0	0	0
Egerer [18]	7	5	2	1	0	0
Farolfi [19]	18	10	6	1	0	0
Heusner [20]	61	27	7	3	2	2
Leyvraz [21]	31	17	7	1	0	0
Leyvraz [4]	86	43	15	5	3	0
Melichar [22]	10	6	2	1	1	1
Peters [25]	100	58	22	6	4	0
Salmon [23]	16	3	1	0	0	0
Siegel [24]	18	11	5	2	0	0

D. Transarterial chemoembolisation (TACE)

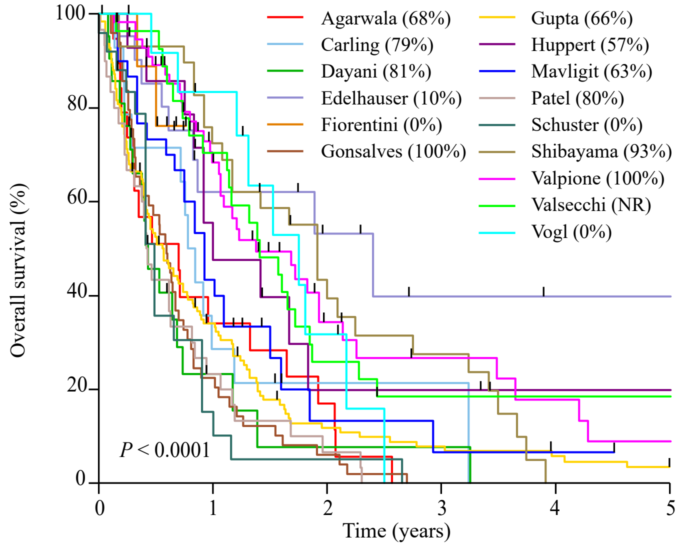

Number at risk

	0	1	2	3	4	5
Agarwala [26]	19	6	3	0	0	0
Carling [1]	14	4	1	1	0	0
Dayani [27]	21	3	1	1	0	0
Edelhauser [28]	21	9	5	2	1	1
Fiorentini [29]	10	0	0	0	0	0
Gonsalves [30]	49	11	3	0	0	0
Gupta [31]	125	39	12	8	5	0
Huppert [32]	14	7	2	2	1	1
Mavligit [33]	30	13	2	1	1	0
Patel [34]	30	7	2	0	0	0
Schuster [35]	25	3	1	0	0	0
Shibayama [36]	29	21	11	7	0	0
Valpione [37]	58	29	10	6	4	2
Valsecchi [38]	27	19	7	4	4	4
Vogl [39]	12	9	3	0	0	0

E. Isolated hepatic perfusion (IHP)

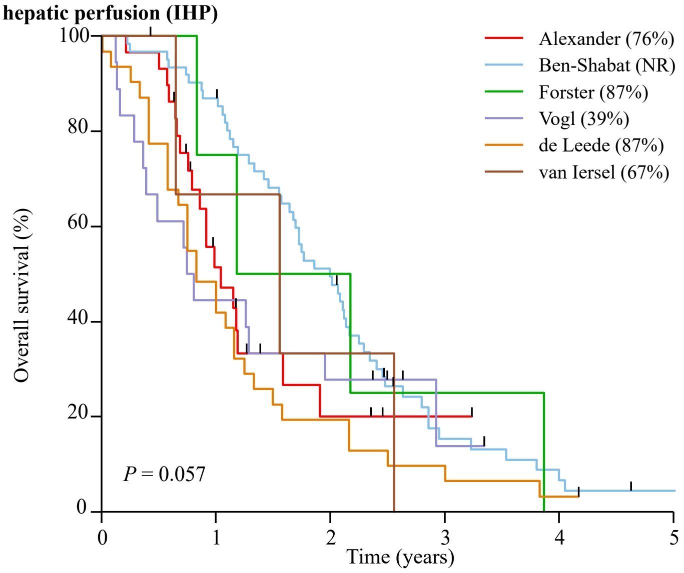

Number at risk

Alexander [40]	29	12	3	1	0	0
Ben-Shabat [41]	61	51	29	7	4	1
Forster [43]	5	3	2	1	0	0
Vogl [45]	18	8	5	1	0	0
de Leede [42]	31	15	6	3	1	0
van Iersel [44]	3	2	1	0	0	0

F. Checkpoint inhibitor (CPI)

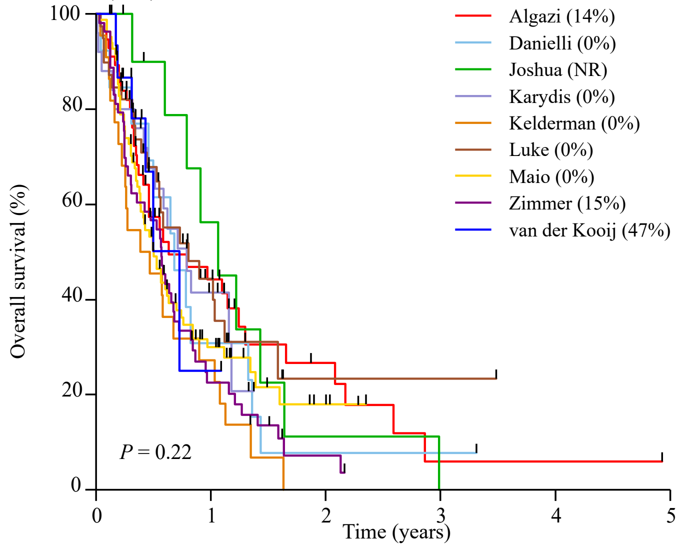

Number at risk

Algazi [46]	56	17	6	1	1	0
Danielli [47]	13	4	1	1	0	0
Joshua [48]	11	5	1	0	0	0
Karydis [49]	25	6	0	0	0	0
Kelderman [50]	22	6	0	0	0	0
Luke [51]	39	10	1	1	0	0
Maio [52]	82	17	2	0	0	0
Zimmer [54]	53	10	2	0	0	0
van der Kooij [53]	17	1	0	0	0	0

G. Protein kinase inhibitor (PKI)

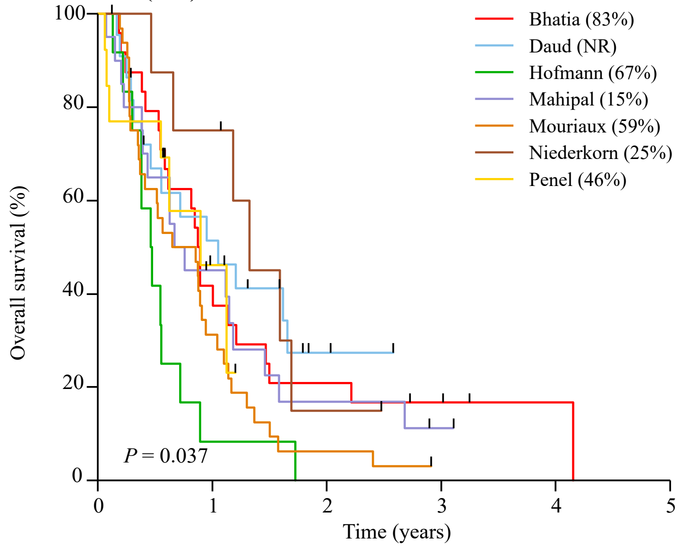

Number at risk

Bhatia [55]	24	10	5	3	1	0
Daud [56]	23	10	2	0	0	0
Hofmann [57]	12	1	0	0	0	0
Mahipal [58]	20	8	3	1	0	0
Mouriaux [59]	32	10	2	0	0	0
Niederkorn [60]	8	6	1	0	0	0
Penel [61]	13	3	0	0	0	0

H. Selective internal radiation therapy (SIRT)

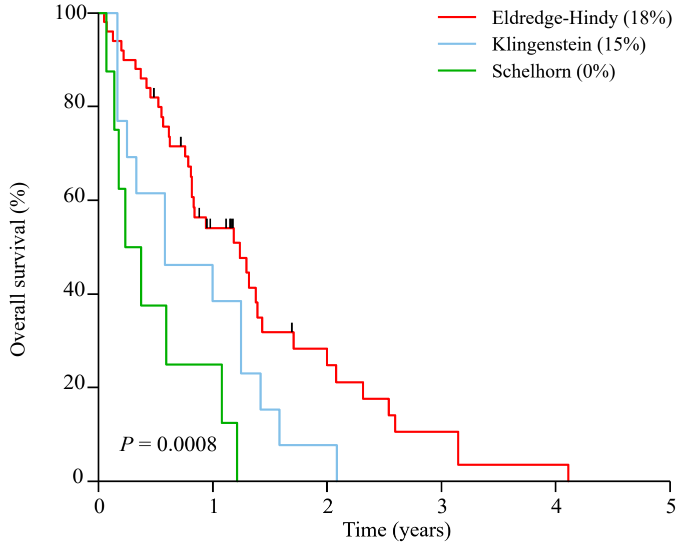

Number at risk

Eldredge-Hindy [62]	50	22	7	3	1	0
Klingenstein [63]	13	6	1	0	0	0
Schelhorn [64]	8	2	0	0	0	0

I. Immunoembolisation (IE)

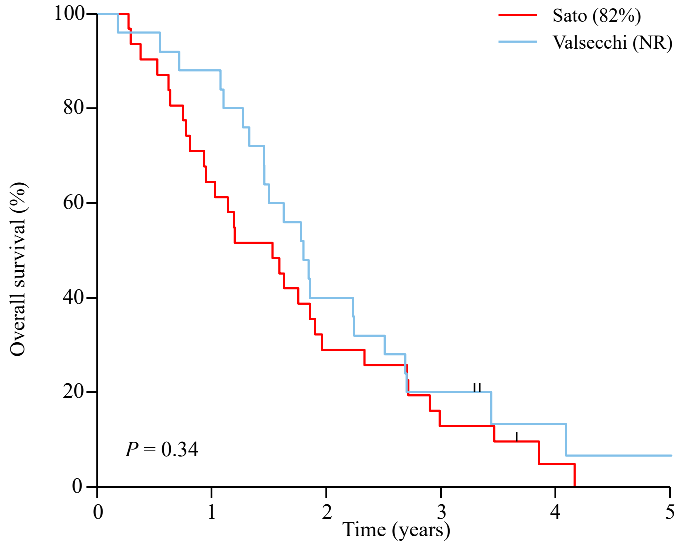

Number at risk						
Sato [65]	31	20	9	4	1	0
Valsecchi [38]	25	22	10	5	2	1

J. Immunosuppressant (IS)

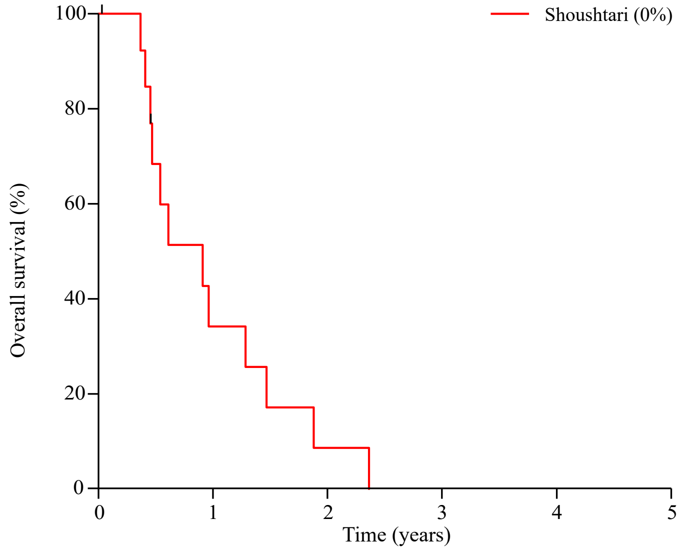

Number at risk						
Shoushtari [66]	14	4	1	0	0	0

K. Liver-directed thermotherapy (LDT)

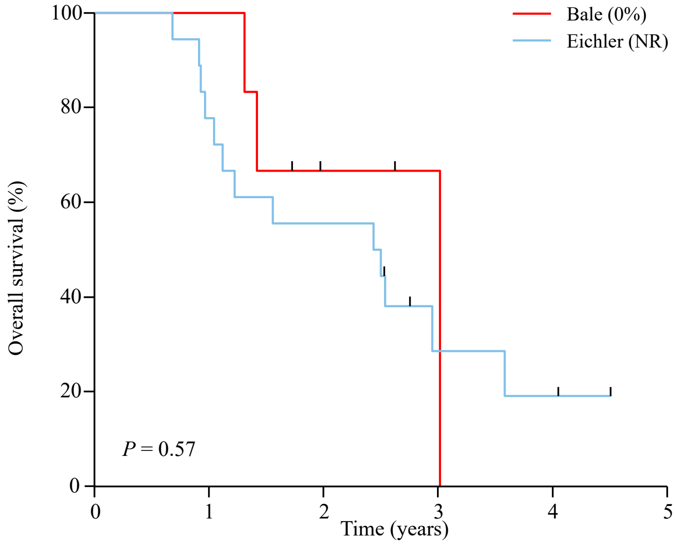

Number at risk

	0	1	2	3	4	5
Bale [67]	6	6	2	1	0	0
Eichler [68]	18	14	10	3	2	0

L. Vaccine

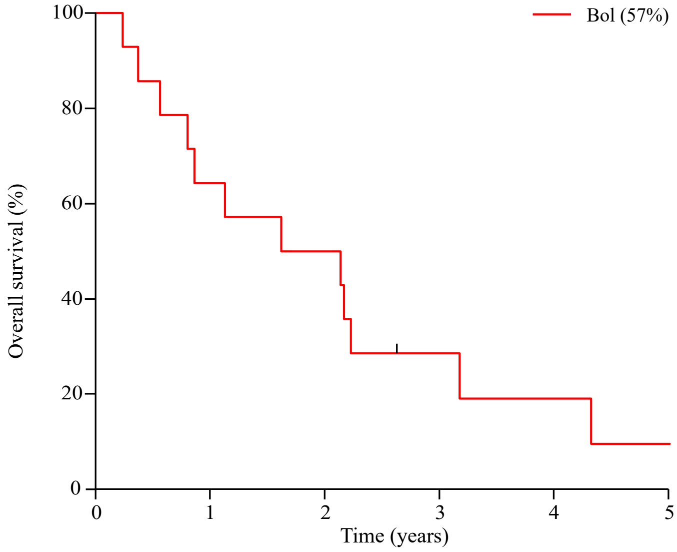

Number at risk

	0	1	2	3	4	5
Bol [69]	14	9	7	3	2	1

M. Surgery

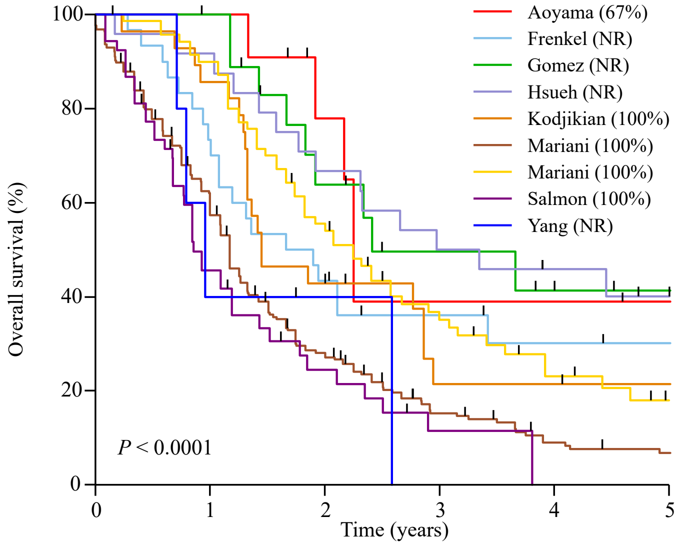

Number at risk

Aoyama [70]	12	11	6	3	3	2
Frenkel [71]	35	22	13	8	5	4
Gomez [72]	18	18	10	6	3	2
Hsueh [73]	24	22	16	12	8	0
Kodjikian [74,75]	28	24	9	4	4	3
Mariani [77]	255	144	59	24	13	8
Mariani [76]	70	63	38	21	10	1
Salmon [23]	53	23	8	3	0	0
Yang [78]	5	2	1	0	0	0

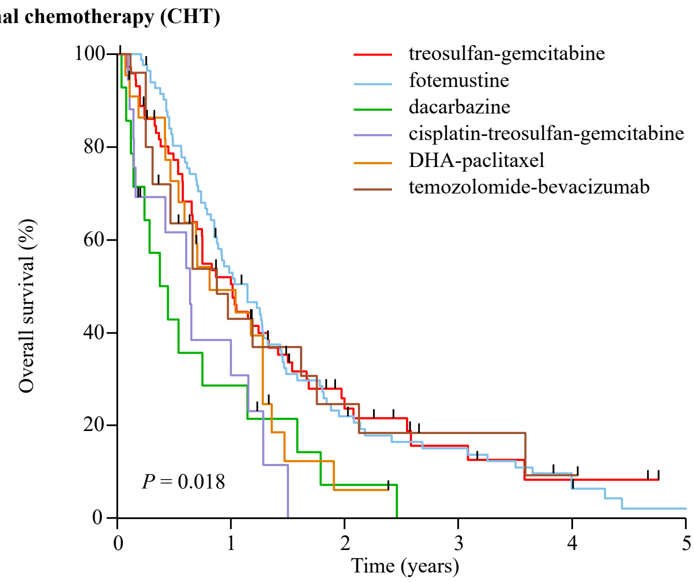

Number at risk

	0	1	2	3	4	5
treosulfan-gemcitabine [2,5,9,10]	72	35	12	5	2	0
fotemustine [4]	85	42	17	11	3	1
dacarbazine [1]	14	4	1	0	0	0
cisplatin-treosulfan-gemcitabine [8]	19	4	0	0	0	0
DHA-paclitaxel [3]	22	10	1	0	0	0
temozolomide-bevacizumab [6]	25	8	4	2	1	0

Fig. S2. Kaplan-Meier plot of overall survival after metastatic uveal melanoma by conventional chemotherapy agents, digitised data for each individual study to display heterogeneity. The spread of the survival curves that derives from differences in study design, inclusion criteria, and case mix may be viewed as an empiric representation of a confidence interval. *P* value was calculated by log-rank test.

A. IS vs CHT

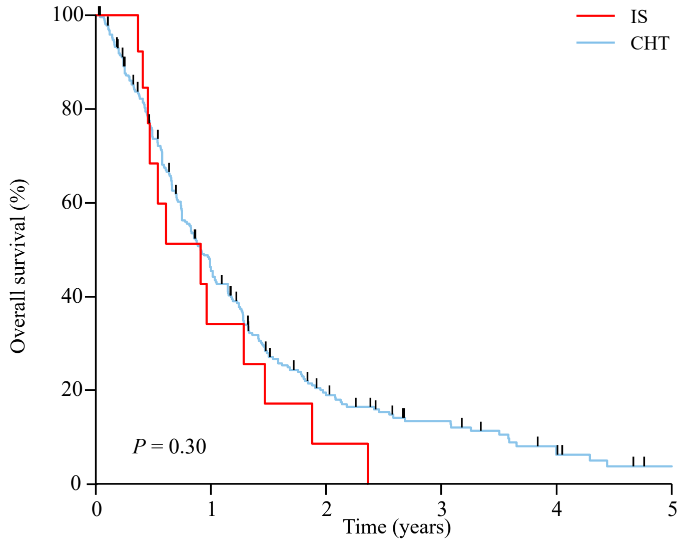

Number at risk

	0	1	2	3	4	5
CHT	272	114	39	19	6	1
IS	14	4	1	0	0	0

B. LDT vs CHT

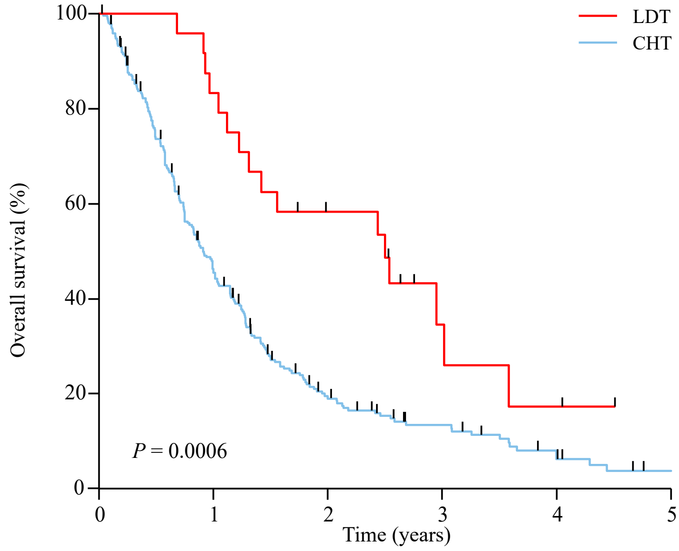

Number at risk

	0	1	2	3	4	5
CHT	272	114	39	19	6	1
LDT	24	20	12	4	2	0

Fig. S3. Kaplan-Meier plots (A-D) of overall survival after metastatic uveal melanoma, pooled data for each treatment modality administered to fewer than 50 patients, compared against conventional chemotherapy. *P* values were calculated by log-rank test.
Abbreviations: CHT, conventional chemotherapy; IS, immunosuppressant; LDT, liver-directed thermotherapy.

C. Vaccine vs CHT

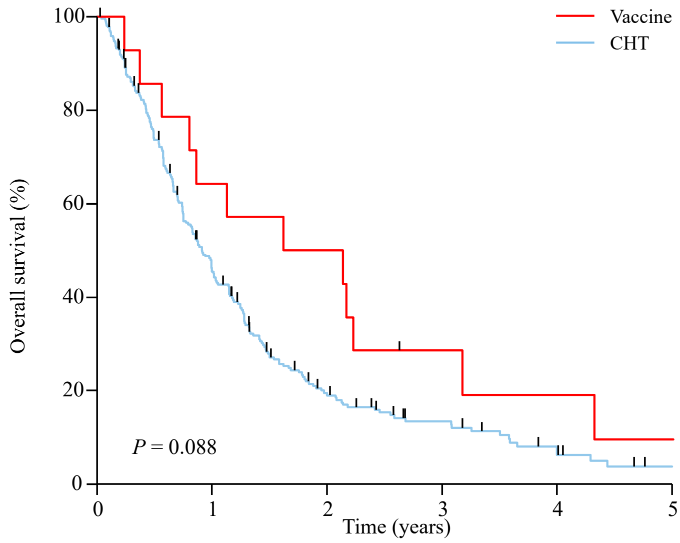

Number at risk

CHT	272	114	39	19	6	1
Vaccine	14	9	7	3	2	1

D. Surgery vs CHT

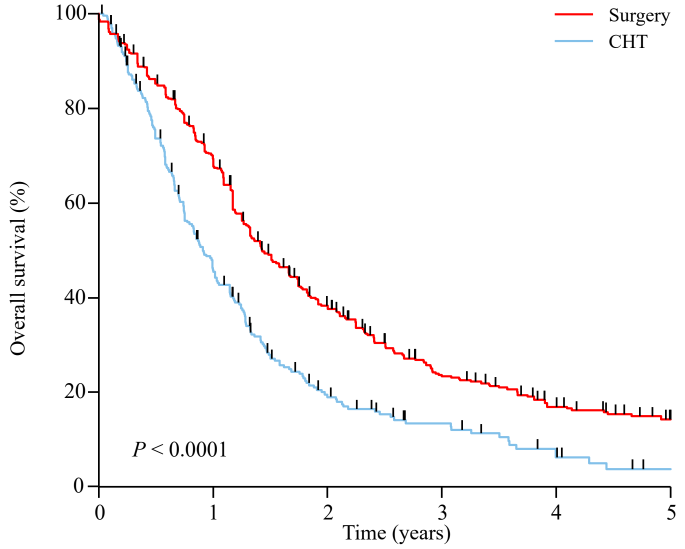

Number at risk

CHT	272	114	39	19	6	1
Surgery	500	329	160	81	46	20

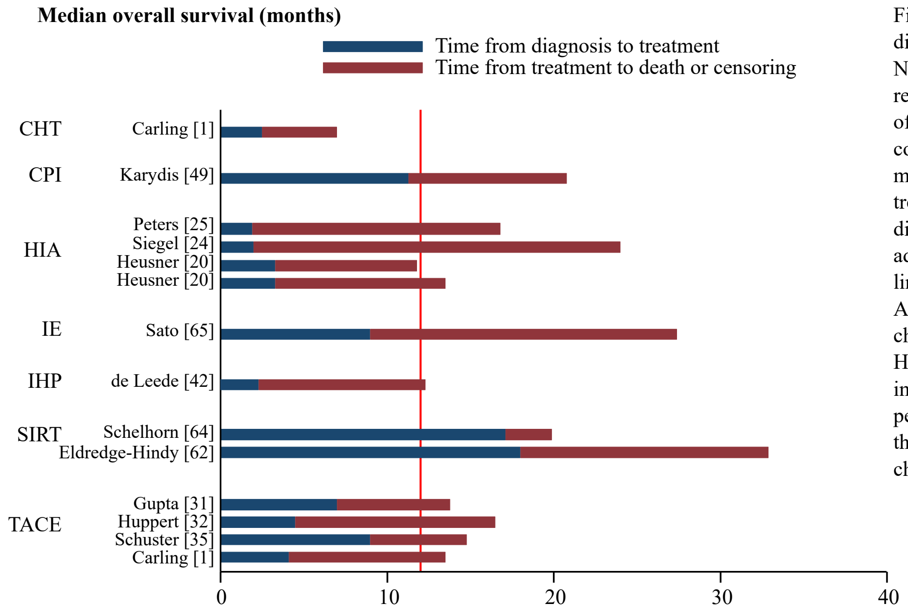

Fig. S4. Components of overall survival from diagnosis of metastasis to death or censoring. Note that the majority of studies would have reported a median overall survival in excess of 12 months (vertical red line) should one consider survival from diagnosis of metastases rather than from initiation of study treatment. The highly variable interval from diagnosis to treatment is a source of bias in addition to the variable percentage of first-line treatments in many reports. Abbreviations: CHT, conventional chemotherapy; CPI, checkpoint inhibitor; HIA, hepatic intra-arterial chemotherapy; IE, immunoembolisation; IHP, isolated hepatic perfusion; SIRT, selective internal radiation therapy; TACE, transarterial chemoembolisation.

IHP vs CHT without Ben-Shabat et al. [41]

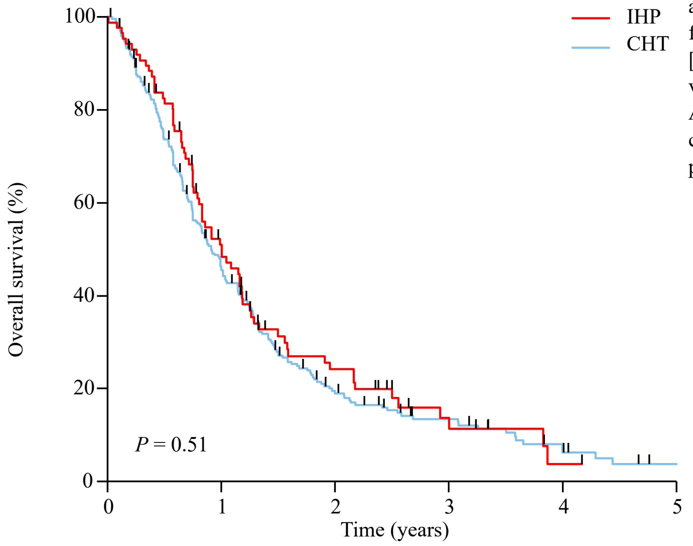

Number at risk

CHT	272	114	39	19	6	1
IHP	86	40	17	6	1	0

Fig. S5. Kaplan-Meier plot of overall survival after metastatic uveal melanoma, pooled data for isolated hepatic perfusion without article [41] against conventional chemotherapy. P-value was calculated by log-rank test. Abbreviations: CHT, conventional chemotherapy; IHP, isolated hepatic perfusion.

Overall survival by decade

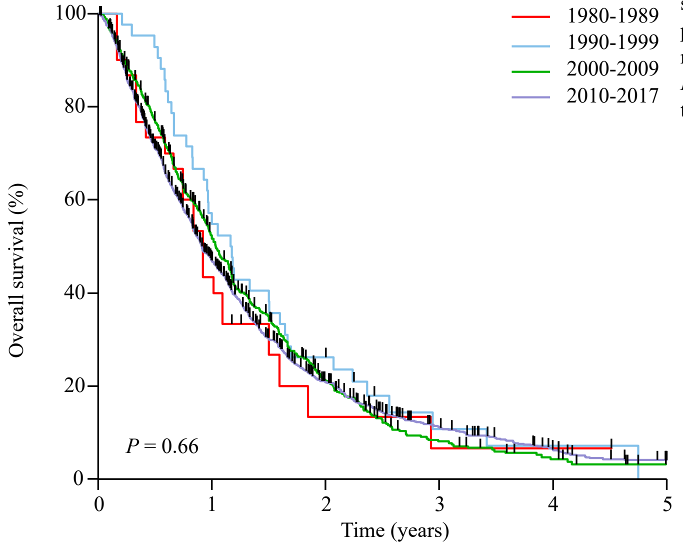

Number at risk

1980-1989	30	13	2	1	1	0
1990-1999	42	24	10	3	1	0
2000-2009	519	236	84	25	10	1
2010-2017	1403	582	214	87	38	14

Fig. S6. Kaplan-Meier plots (A-B) of overall survival after metastatic uveal melanoma, pooled data by decade. All treatment modalities were included except for surgery. *P* value was calculated by log-rank test for trend.

A. Overall survival by percentage of first-line treatments

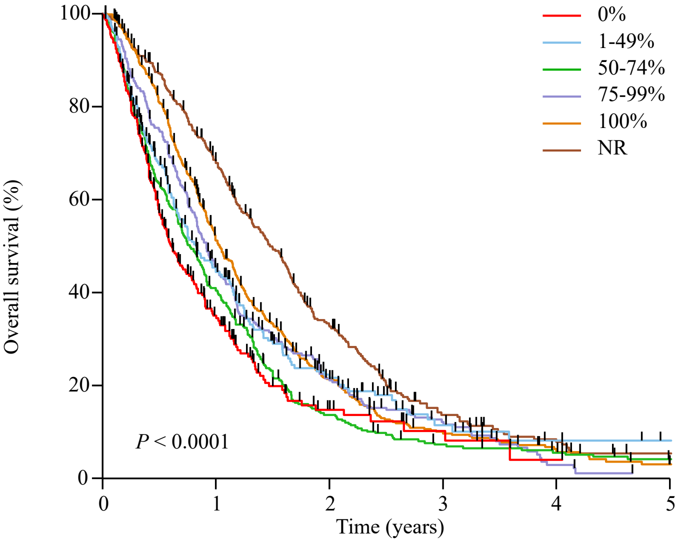

Fig. S7. Kaplan-Meier plots (A-B) of overall survival after metastatic uveal melanoma, pooled data by percentage of first-line treatments. All treatment modalities were included except for surgery. *P* values were calculated by log-rank test for trend. Abbreviation: NR, not reported (curve not included when calculating the *P* value).

Number at risk

0%	275	67	13	5	1	0
1-49%	253	89	31	9	3	1
50-74%	357	140	42	19	12	5
75-99%	269	117	48	23	4	0
100%	510	232	84	32	15	3
NR	330	210	92	28	15	6

B. Overall survival by percentage of first-line treatments

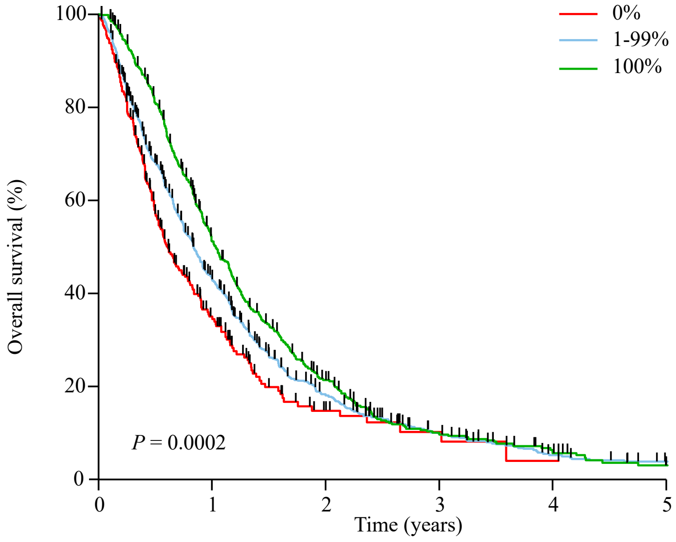

Number at risk

0%	275	67	13	5	1	0
1-99%	879	346	121	51	19	6
100%	510	232	84	32	15	3

References

1 Carling U, Dorenberg EJ, Haugvik SP, Eide NA, Berntzen DT, Edwin B, *et al.* Transarterial chemoembolization of liver metastases from uveal melanoma using irinotecan-loaded beads: treatment response and complications. *Cardiovasc Intervent Radiol* 2015; **38**:1532-1541.

2 Corrie PG, Shaw J, Spanswick VJ, Sehmbi R, Jonson A, Mayer A, *et al*. Phase I trial combining gemcitabine and treosulfan in advanced cutaneous and uveal melanoma patients. *Br J Cancer* 2005; **92**:1997-2003.

3 Homsi J, Bedikian AY, Papadopoulos NE, Kim KB, Hwu WJ, Mahoney SL, et al. Phase 2 open-label study of weekly docosahexaenoic acid-paclitaxel in patients with metastatic uveal melanoma. *Melanoma Res* 2010; **20**:507-510.

4 Leyvraz S, Piperno-Neumann S, Suciu S, Baurain JF, Zdzienicki M, Testori A, *et al.* Hepatic intra-arterial versus intravenous fotemustine in patients with liver metastases from uveal melanoma (EORTC 18021): a multicentric randomized trial. *Ann Oncol* 2014; **25**:742-746.

5 Pföhler C, Cree IA, Ugurel S, Kuwert C, Haass N, Neuber K, et al. Treosulfan and gemcitabine in metastatic uveal melanoma patients: results of a multicenter feasibility study. *Anticancer Drugs* 2003; **14**:337-340.

6 Piperno-Neumann S, Diallo A, Etienne-Grimaldi MC, Bidard FC, Rodrigues M, Plancher C, *et al.* Phase II trial of bevacizumab in combination with temozolomide as first-line treatment in patients with metastatic uveal melanoma. *Oncologist* 2016; **21**:281-282.

7 Pons F, Plana M, Caminal JM, Pera J, Fernandes I, Perez J, *et al.* Metastatic uveal melanoma: is there a role for conventional chemotherapy? - A single center study based on 58 patients. *Melanoma Res* 2011; **21**:217-222.

8 Schmittel A, Scheulen ME, Bechrakis NE, Strumberg D, Baumgart J, Bornfeld N, *et al.* Phase II trial of cisplatin, gemcitabine and treosulfan in patients with metastatic uveal melanoma. *Melanoma Res* 2005; **15**:205-207.

9 Schmittel A, Schuster R, Bechrakis NE, Siehl JM, Foerster MH, Thiel E, *et al.* A two-cohort phase II clinical trial of gemcitabine plus treosulfan in patients with metastatic uveal melanoma. *Melanoma Res* 2005; **15**:447-451.

10 Terheyden P, Bröcker EB, Becker JC. Clinical evaluation of in vitro chemosensitivity testing: the example of uveal melanoma. *J Cancer Res Clin Oncol* 2004; **130**:395-399.

11 Becker JC, Terheyden P, Kämpgen E, Wagner S, Neumann C, Schadendorf D, *et al*. Treatment of disseminated ocular melanoma with sequential fotemustine, interferon alpha, and interleukin 2. *Br J Cancer* 2002; **87**:840-845.

12 Kivelä T, Suciu S, Hansson J, Kruit WH, Vuoristo MS, Kloke O, *et al.* Bleomycin, vincristine, lomustine and dacarbazine (BOLD) in combination with recombinant interferon alpha-2b for metastatic uveal melanoma. *Eur J Cancer* 2003; **39**:1115-1120.

13 Pyrhönen S, Hahka-Kemppinen M, Muhonen T, Nikkanen V, Eskelin S, Summanen P, *et al.* Chemoimmunotherapy with bleomycin, vincristine, lomustine, dacarbazine (BOLD), and human leukocyte interferon for metastatic uveal melanoma. *Cancer* 2002; **95**:2366-2372.

14 Solti M, Berd D, Mastrangelo MJ, Sato T. A pilot study of low-dose thalidomide and interferon alpha-2b in patients with metastatic melanoma who failed prior treatment. *Melanoma Res* 2007; **17**:225-231.

15 Terheyden P, Kämpgen E, Rünger TM, Bröcker EB, Becker JC. Immunochemotherapie des metastasierenden Uveamelanoms mit Interferon-alpha 2b, Interleukin-2 und Fotemustin. Kasuistiken und Literaturübersicht. *Hautarzt* 1998; **49**:770-773.

16 Vihinen PP, Hernberg M, Vuoristo MS, Tyynelä K, Laukka M, Lundin J, *et al.* A phase II trial of bevacizumab with dacarbazine and daily low-dose interferon-alpha2a as first line treatment in metastatic melanoma. *Melanoma Res* 2010; **20**:318-325.

17 Cantore M, Fiorentini G, Aitini E, Davitti B, Cavazzini G, Rabbi C, *et al.* Intra-arterial hepatic carboplatin-based chemotherapy for ocular melanoma metastatic to the liver. Report of a phase II study. *Tumori* 1994; **80**:37-39.

18 Egerer G, Lehnert T, Max R, Naeher H, Keilholz U, Ho AD. Pilot study of hepatic intraarterial fotemustine chemotherapy for liver metastases from uveal melanoma: a single-center experience with seven patients. *Int J Clin Oncol* 2001; **6**:25-28.

19 Farolfi A, Ridolfi L, Guidoboni M, Milandri C, Calzolari F, Scarpi E, *et al.* Liver metastases from melanoma: hepatic intra-arterial chemotherapy. A retrospective study. *J Chemother* 2011; **23**:300-305.

20 Heusner TA, Antoch G, Wittkowski-Sterczewski A, Ladd SC, Forsting M, Verhagen R, *et al*. Transarterial hepatic chemoperfusion of uveal melanoma metastases: survival and response to treatment. *Rofo* 2011; **183**:1151-1160.

21 Leyvraz S, Spataro V, Bauer J, Pampallona S, Salmon R, Dorval T, *et al*. Treatment of ocular melanoma metastatic to the liver by hepatic arterial chemotherapy. *J Clin Oncol* 1997; **15**:2589-2595.

22 Melichar B, Vobořil Z, Lojik M, Krajina A. Liver metastases from uveal melanoma: clinical experience of hepatic arterial infusion of cisplatin, vinblastine and dacarbazine. *Hepatogastroenterology* 2009; **56**:1157-1162.

23 Salmon RJ, Levy C, Plancher C, Dorval T, Desjardins L, Leyvraz S, *et al.* Treatment of liver metastases from uveal melanoma by combined surgery-chemotherapy. *Eur J Surg Oncol* 1998; **24**:127-130.

24 Siegel R, Hauschild A, Kettelhack C, Kähler KC, Bembenek A, Schlag PM. Hepatic arterial fotemustine chemotherapy in patients with liver metastases from cutaneous melanoma is as effective as in ocular melanoma. *Eur J Surg Oncol* 2007; **33**:627-632.
25 Peters S, Voelter V, Zografos L, Pampallona S, Popescu R, Gillet M, *et al.* Intra-arterial hepatic fotemustine for the treatment of liver metastases from uveal melanoma: experience in 101 patients. *Ann Oncol* 2006; **17**:578-583.
26 Agarwala SS, Panikkar R, Kirkwood JM. Phase I/II randomized trial of intrahepatic arterial infusion chemotherapy with cisplatin and chemoembolization with cisplatin and polyvinyl sponge in patients with ocular melanoma metastatic to the liver. *Melanoma Res* 2004; **14**:217-222.
27 Dayani PN, Gould JE, Brown DB, Sharma KV, Linette GP, Harbour JW. Hepatic metastasis from uveal melanoma: angiographic pattern predictive of survival after hepatic arterial chemoembolization. *Arch Ophthalmol* 2009; **127**:628-632.
28 Edelhauser G, Schicher N, Berzaczy D, Beitzke D, Höeller C, Lammer J, *et al.* Fotemustine chemoembolization of hepatic metastases from uveal melanoma: a retrospective single-center analysis. *American journal of roentgenology* 2012; **199**:1387-1392.
29 Fiorentini G, Aliberti C, Del Conte A, Tilli M, Rossi S, Ballardini P, *et al.* Intra-arterial hepatic chemoembolization (TACE) of liver metastases from ocular melanoma with slow-release irinotecan-eluting beads. Early results of a phase II clinical study. *In Vivo* 2009; **23**:131-137.
30 Gonsalves CF, Eschelman DJ, Thornburg B, Frangos A, Sato T. Uveal melanoma metastatic to the liver: chemoembolization with 1,3-bis-(2-chloroethyl)-1-nitrosourea. *American journal of roentgenology* 2015; **205**:429-433.
31 Gupta S, Bedikian AY, Ahrar J, Ensor J, Ahrar K, Madoff DC, *et al.* Hepatic artery chemoembolization in patients with ocular melanoma metastatic to the liver: response, survival, and prognostic factors. *Am J Clin Oncol* 2010; **33**:474-480.
32 Huppert PE, Fierlbeck G, Pereira P, Schanz S, Duda SH, Wietholtz H, *et al.* Transarterial chemoembolization of liver metastases in patients with uveal melanoma. *Eur J Radiol* 2010; **74**:e38-44.
33 Mavligit GM, Charnsangavej C, Carrasco CH, Patt YZ, Benjamin RS, Wallace S. Regression of ocular melanoma metastatic to the liver after hepatic arterial chemoembolization with cisplatin and polyvinyl sponge. *JAMA* 1988; **260**:974-976.
34 Patel K, Sullivan K, Berd D, Mastrangelo MJ, Shields CL, Shields JA, *et al.* Chemoembolization of the hepatic artery with BCNU for metastatic uveal melanoma: results of a phase II study. *Melanoma Res* 2005; **15**:297-304.
35 Schuster R, Lindner M, Wacker F, Krössin M, Bechrakis N, Foerster MH, *et al.* Transarterial chemoembolization of liver metastases from uveal melanoma after failure of systemic therapy: toxicity and outcome. *Melanoma Res* 2010; **20**:191-196.
36 Shibayama Y, Namikawa K, Sone M, Takahashi A, Tsutsumida A, Sugawara S, *et al.* Efficacy and toxicity of transarterial chemoembolization therapy using cisplatin and gelatin sponge in patients with liver metastases from uveal melanoma in an Asian population. *Int J Clin Oncol* 2017.
37 Valpione S, Aliberti C, Parrozzani R, Bazzi M, Pigozzo J, Midena E, *et al.* A retrospective analysis of 141 patients with liver metastases from uveal melanoma: a two-cohort study comparing transarterial chemoembolization with CPT-11 charged microbeads and historical treatments. *Melanoma Res* 2015; **25**:164-168.
38 Valsecchi ME, Terai M, Eschelman DJ, Gonsalves CF, Chervoneva I, Shields JA, *et al.* Double-blinded, randomized phase II study using embolization with or without granulocyte-macrophage colony-stimulating factor in uveal melanoma with hepatic metastases. *J Vasc Interv Radiol* 2015; **26**:523-532.e522.
39 Vogl T, Eichler K, Zangos S, Herzog C, Hammerstingl R, Balzer J, *et al.* Preliminary experience with transarterial chemoembolization (TACE) in liver metastases of uveal malignant melanoma: local tumor control and survival. *J Cancer Res Clin Oncol* 2007; **133**:177-184.
40 Alexander HR, Jr., Libutti SK, Pingpank JF, Steinberg SM, Bartlett DL, Helsabeck C, *et al.* Hyperthermic isolated hepatic perfusion using melphalan for patients with ocular melanoma metastatic to liver. *Clin Cancer Res* 2003; **9**:6343-6349.
41 Ben-Shabat I, Belgrano V, Ny L, Nilsson J, Lindnér P, Olofsson Bagge R. Long-term follow-up evaluation of 68 patients with uveal melanoma liver metastases treated with isolated hepatic perfusion. *Ann Surg Oncol* 2016; **23**:1327-1334.
42 de Leede EM, Burgmans MC, Kapiteijn E, Luyten GP, Jager MJ, Tijl FG, *et al.* Isolated (hypoxic) hepatic perfusion with high-dose chemotherapy in patients with unresectable liver metastases of uveal melanoma: results from two experienced centres. *Melanoma Res* 2016; **26**:588-594.
43 Forster MR, Rashid OM, Perez MC, Choi J, Chaudhry T, Zager JS. Chemosaturation with percutaneous hepatic perfusion for unresectable metastatic melanoma or sarcoma to the liver: a single institution experience. *J Surg Oncol* 2014; **109**:434-439.
44 van Iersel LB, de Leede EM, Vahrmeijer AL, Tijl FG, den Hartigh J, Kuppen PJ, *et al.* Isolated hepatic perfusion with oxaliplatin combined with 100 mg melphalan in patients with metastases confined to the liver: A phase I study. *Eur J Surg Oncol* 2014; **40**:1557-1563.

45 Vogl TJ, Koch SA, Lotz G, Gebauer B, Willinek W, Engelke C, *et al.* Percutaneous isolated hepatic perfusion as a treatment for isolated hepatic metastases of uveal melanoma: patient outcome and safety in a multi-centre study. *Cardiovasc Intervent Radiol* 2017.

46 Algazi AP, Tsai KK, Shoushtari AN, Munhoz RR, Eroglu Z, Piulats JM, *et al*. Clinical outcomes in metastatic uveal melanoma treated with PD-1 and PD-L1 antibodies. *Cancer* 2016; **122**:3344-3353.

47 Danielli R, Ridolfi R, Chiarion-Sileni V, Queirolo P, Testori A, Plummer R, *et al*. Ipilimumab in pretreated patients with metastatic uveal melanoma: safety and clinical efficacy. *Cancer Immunol Immunother* 2012; **61**:41-48.

48 Joshua AM, Monzon JG, Mihalcioiu C, Hogg D, Smylie M, Cheng T. A phase 2 study of tremelimumab in patients with advanced uveal melanoma. *Melanoma Res* 2015; **25**:342-347.

49 Karydis I, Chan PY, Wheater M, Arriola E, Szlosarek PW, Ottensmeier CH. Clinical activity and safety of pembrolizumab in ipilimumab pre-treated patients with uveal melanoma. *Oncoimmunology* 2016; **5**:e1143997.

50 Kelderman S, van der Kooij MK, van den Eertwegh AJ, Soetekouw PM, Jansen RL, van den Brom RR, *et al*. Ipilimumab in pretreated metastastic uveal melanoma patients. Results of the Dutch Working Group on Immunotherapy of Oncology (WIN-O). *Acta Oncol* 2013; **52**:1786-1788.

51 Luke JJ, Callahan MK, Postow MA, Romano E, Ramaiya N, Bluth M, *et al*. Clinical activity of ipilimumab for metastatic uveal melanoma: a retrospective review of the Dana-Farber Cancer Institute, Massachusetts General Hospital, Memorial Sloan-Kettering Cancer Center, and University Hospital of Lausanne experience. *Cancer* 2013; **119**:3687-3695.

52 Maio M, Danielli R, Chiarion-Sileni V, Pigozzo J, Parmiani G, Ridolfi R, *et al*. Efficacy and safety of ipilimumab in patients with pre-treated, uveal melanoma. *Ann Oncol* 2013; **24**:2911-2915.

53 van der Kooij MK, Joosse A, Speetjens FM, Hospers GA, Bisschop C, de Groot JW, *et al*. Anti-PD1 treatment in metastatic uveal melanoma in the Netherlands. *Acta Oncol* 2017; **56**:101-103.

54 Zimmer L, Vaubel J, Mohr P, Hauschild A, Utikal J, Simon J, *et al*. Phase II DeCOG-study of ipilimumab in pretreated and treatment-naive patients with metastatic uveal melanoma. *PLoS One* 2015; **10**:e0118564.

55 Bhatia S, Moon J, Margolin KA, Weber JS, Lao CD, Othus M, *et al*. Phase II trial of sorafenib in combination with carboplatin and paclitaxel in patients with metastatic uveal melanoma: SWOG S0512. *PLoS One* 2012; **7**:e48787.

56 Daud A, Kluger HM, Kurzrock R, Schimmoller F, Weitzman AL, Samuel TA, *et al*. Phase II randomised discontinuation trial of the MET/VEGF receptor inhibitor cabozantinib in metastatic melanoma. *Br J Cancer* 2017; **116**:432-440.

57 Hofmann UB, Kauczok-Vetter CS, Houben R, Becker JC. Overexpression of the KIT/SCF in uveal melanoma does not translate into clinical efficacy of imatinib mesylate. *Clin Cancer Res* 2009; **15**:324-329.

58 Mahipal A, Tijani L, Chan K, Laudadio M, Mastrangelo MJ, Sato T. A pilot study of sunitinib malate in patients with metastatic uveal melanoma. *Melanoma Res* 2012; **22**:440-446.

59 Mouriaux F, Servois V, Parienti JJ, Lesimple T, Thyss A, Dutriaux C, *et al*. Sorafenib in metastatic uveal melanoma: efficacy, toxicity and health-related quality of life in a multicentre phase II study. *Br J Cancer* 2016; **115**:20-24.

60 Niederkorn A, Wackernagel W, Artl M, Schwantzer G, Aigner B, Richtig E. Response of patients with metastatic uveal melanoma to combined treatment with fotemustine and sorafenib. *Acta Ophthalmol* 2014; **92**:e696-697.

61 Penel N, Delcambre C, Durando X, Clisant S, Hebbar M, Negrier S, *et al*. O-Mel-Inib: a Cancero-pole Nord-Ouest multicenter phase II trial of high-dose imatinib mesylate in metastatic uveal melanoma. *Invest New Drugs* 2008; **26**:561-565.

62 Eldredge-Hindy H, Ohri N, Anne PR, Eschelman D, Gonsalves C, Intenzo C, *et al*. Yttrium-90 microsphere brachytherapy for liver metastases from uveal melanoma: clinical outcomes and the predictive value of fluorodeoxyglucose positron emission tomography. *Am J Clin Oncol* 2016; **39**:189-195.

63 Klingenstein A, Haug AR, Zech CJ, Schaller UC. Radioembolization as locoregional therapy of hepatic metastases in uveal melanoma patients. *Cardiovasc Intervent Radiol* 2013; **36**:158-165.

64 Schelhorn J, Richly H, Ruhlmann M, Lauenstein TC, Theysohn JM. A single-center experience in radioembolization as salvage therapy of hepatic metastases of uveal melanoma. *Acta Radiol Open* 2015; **4**:2047981615570417.

65 Sato T, Eschelman DJ, Gonsalves CF, Terai M, Chervoneva I, McCue PA, *et al*. Immunoembolization of malignant liver tumors, including uveal melanoma, using granulocyte-macrophage colony-stimulating factor. *J Clin Oncol* 2008; **26**:5436-5442.

66 Shoushtari AN, Ong LT, Schoder H, Singh-Kandah S, Abbate KT, Postow MA, *et al*. A phase 2 trial of everolimus and pasireotide long-acting release in patients with metastatic uveal melanoma. *Melanoma Res* 2016; **26**:272-277.

67 Bale R, Schullian P, Schmuth M, Widmann G, Jaschke W, Weinlich G. Stereotactic radiofrequency ablation for metastatic melanoma to the liver. *Cardiovasc Intervent Radiol* 2016; **39**:1128-1135.

68 Eichler K, Zangos S, Gruber-Rouh T, Vogl TJ, Mack MG. MR-guided laser-induced thermotherapy (LITT) in patients with liver metastases of uveal melanoma. *J Eur Acad Dermatol Venereol* 2014; **28**:1756-1760.

69 Bol KF, Mensink HW, Aarntzen EH, Schreibelt G, Keunen JE, Coulie PG, *et al*. Long overall survival after dendritic cell vaccination in metastatic uveal melanoma patients. *Am J Ophthalmol* 2014; **158**:939-947.

70 Aoyama T, Mastrangelo MJ, Berd D, Nathan FE, Shields CL, Shields JA, *et al*. Protracted survival after resection of metastatic uveal melanoma. *Cancer* 2000; **89**:1561-1568.

71 Frenkel S, Nir I, Hendler K, Lotem M, Eid A, Jurim O, *et al*. Long-term survival of uveal melanoma patients after surgery for liver metastases. *Br J Ophthalmol* 2009; **93**:1042-1046.

72 Gomez D, Wetherill C, Cheong J, Jones L, Marshall E, Damato B, *et al*. The Liverpool uveal melanoma liver metastases pathway: outcome following liver resection. *J Surg Oncol* 2014; **109**:542-547.

73 Hsueh EC, Essner R, Foshag LJ, Ye X, Wang HJ, Morton DL. Prolonged survival after complete resection of metastases from intraocular melanoma. *Cancer* 2004; **100**:122-129.

74 Kodjikian L, Grange JD, Baldo S, Baillif S, Garweg JG, Rivoire M. Prolonged survival after resection of liver metastases from uveal melanoma and intra-arterial chemotherapy. *Graefes Arch Clin Exp Ophthalmol* 2005; **243**:622-624.

75 Rivoire M, Kodjikian L, Baldo S, Kaemmerlen P, Négrier S, Grange JD. Treatment of liver metastases from uveal melanoma. *Ann Surg Oncol* 2005; **12**:422-428.

76 Mariani P, Almubarak MM, Kollen M, Wagner M, Plancher C, Audollent R, *et al*. Radiofrequency ablation and surgical resection of liver metastases from uveal melanoma. *Eur J Surg Oncol* 2016; **42**:706-712.

77 Mariani P, Piperno-Neumann S, Servois V, Berry MG, Dorval T, Plancher C, *et al*. Surgical management of liver metastases from uveal melanoma: 16 years' experience at the Institut Curie. *Eur J Surg Oncol* 2009; **35**:1192-1197.

78 Yang XY, Xie F, Tao R, Li AJ, Wu MC. Treatment of liver metastases from uveal melanoma: a retrospective single-center analysis. *Hepatobiliary Pancreat Dis Int* 2013; **12**:602-606.
