# Supplementary material for: Overall survival after treatment for metastatic uveal melanoma: a systematic review and meta-analysis
Source: Melanoma Res. 2019 Jan 16;29(6):561–8. doi: 10.1097/CMR.0000000000000575 (PMC6887637; doi:10.1097/CMR.0000000000000575)
Supplement: Supplementary file 5 [file mr-29-561-s005.pdf]

Supplemental Digital Content 5 – **Proposed guidelines for reporting studies on treatment for metastatic uveal melanoma based on our experience in extracting the data for the systematic review and meta-analysis:**

- anatomical site clearly specified, “conjunctival” or “uveal”; more precisely “choroidal”, “ciliary body”, or “iris”; and not “ocular”
- survival analysis carried out by the Kaplan-Meier method
- median interval from diagnosis of metastases to initiation of treatment reported
- beginning of the survival period, e.g. initiation of treatment, diagnosis of the metastases
- censorings along with a at-risk table provided
- median, rather than mean survival reported, including the method by which it was obtained, e.g. arithmetically, actuarial method, Kaplan-Meier method
- survival rate reported at key time points, e.g. 1-year, 2-year, 3-year
- patuent-level data provided through a data repository, inclusing available prognostic factors, e.g. staging, performance status, liver function, genetic alterations.
