# Supplementary material for: Overall survival after treatment for metastatic uveal melanoma: a systematic review and meta-analysis
Source: Melanoma Res. 2019 Jan 16;29(6):561–8. doi: 10.1097/CMR.0000000000000575 (PMC6887637; doi:10.1097/CMR.0000000000000575)
Supplement: Supplementary file 6 [file mr-29-561-s006.pdf]

Supplemental Digital Content 6 – Overall survival benchmark

Constructing the historical control overall survival distribution for a phase II trial

The historical control curve is given by:

$$\bar{S}(t) = \frac{1}{n}\sum_{i=1}^{n} S_i\,(t)$$

where

$$S_i(t) = [S_0(t)]^{exp(SUM_i)}$$

$S_0(t)$ is given in the Supplementary Excel File, Supplemental Digital Content 7, based on 78 articles, 2494 patients.

n is number of the patients in the phase II trial.

At the time of analysis of the new phase II trial, the survival curve of this trial based on *n* patients is compared with the historical survival curve [1]. Somewhat arbitrarily the endpoint was chosen to be one year. As suggested by Korn *et al.* [1] if *P*-value is less than 0.01, the new drug can be pursued further.

References

1 Korn EL, Liu P-Y, Lee SJ, Chapman J-AW, Niedzwiecki D, Suman VJ, *et al*. Meta-analysis of phase II cooperative group trials in metastatic stage IV melanoma to determine progression-free and overall survival benchmarks for future phase II trials. *J Clin Oncol* 2008; **26**:527-534.
